# Cold Storage Effects on Fitness of the Whitefly Parasitoids *Encarsia sophia* and *Eretmocerus hayati*

**DOI:** 10.3390/insects11070428

**Published:** 2020-07-09

**Authors:** Dawit Kidane, Marco Ferrante, Xiao-Ming Man, Wan-Xue Liu, Fang-Hao Wan, Nian-Wan Yang

**Affiliations:** 1 State Key Laboratory for Biology of Plant Diseases and Insect Pests, Institute of Plant Protection, Chinese Academy of Agricultural Sciences, Beijing 100193, China; dawit.kidane@mu.edu.et (D.K.); 13655423193@163.com (X.-M.M.); liuwanxue@caas.cn (W.-X.L.); wanfanghao@caas.cn (F.-H.W.); 2 Department of Biology, College of Natural and Computational Sciences, Mekelle University, Tigray 7000, Ethiopia; 3 CE3C-Centre for Ecology, Evolution and Environmental Changes, Azorean Biodiversity Group, Faculty of Agricultural and Environmental Sciences, University of the Azores, PT-9700-042 Angra do Heroísmo, Portugal; marco.ferrante@live.it; 4 Guangdong Laboratory of Lingnan Modern Agriculture, Shenzhen, Genome Analysis Laboratory of the Ministry of Agriculture and Rural Area, Agricultural Genomics Institute at Shenzhen, Chinese Academy of Agricultural Sciences, Shenzhen 518120, China; 5 College of Plant Health and Medicine, Qingdao Agricultural University, Qingdao 266109, China

**Keywords:** fluctuating temperature, parasitoid, Hymenoptera, *Aphelinidae*, *Bemisia tabaci*, *Aleyrodidae*

## Abstract

Successful biological control of the whitefly *Bemisia tabaci* involves the mass rearing of biocontrol agents in large numbers for field release. Cold storage of the biocontrol agents is often necessary to provide a sufficient number of biocontrol agents during an eventual pest outbreak. In this study, the fitness of two whitefly parasitoids *Encarsia sophia* Girault and Dodd (Hymenoptera: Aphelinidae) and *Eretmocerus hayati* Zolnerowich and Rose (Hymenoptera: Aphelinidae) was evaluated under fluctuating cold storage temperatures. The emergence rate of old pupae of either species was not affected when stored at 12, 10, 8 and 6 °C for 1 week. Cold storage had no effect on the longevity of the emerging adult *En. sophia* except young pupae stored at 4 °C, while *Er. hayati* was negatively affected after 2 weeks of storage time at all temperatures. Parasitism by adults emerging from older pupae stored at 12 °C for 1 week was equivalent to the control. Combined with the results for the emergence time, we suggest that the old pupal stage of *En. sophia* and *Er. hayati* could be stored at 12 and 10 °C, respectively (transferred every 22 h to 26 ± 1 °C for 2 h), for 1 week, with no or little adverse effect.

## 1. Introduction

Cold storage is an important tool in the practice of mass rearing biocontrol agents [1]. It aims to ensure the availability of a sufficient number of natural enemies when they are needed [2,3], allows for synchronized field releases of natural enemies during the critical stages of pest outbreaks [4,5], is a valuable method for increasing the shelf-life of mass-reared insect parasitoids [2] and could improve the continuous rearing practices of insects for pest management [6]. Biocontrol agents are normally stored in the pupal stage, which then develop inside the host insects [1,7,8]. Cold storage of biocontrol agents is usually performed under a constant low temperature [1]. However, insects are exposed to changing temperatures in nature, and it is important to consider the various effects of both constant and fluctuating temperatures [9]. The response of insects can differ greatly when exposed to variable temperatures compared to constant ones [9,10,11,12]. Cold storage of parasitoids under fluctuating cold temperatures can have a positive effect on their fitness [2,9,13,14,15]. This highlights the need for more investigations into the effect of variable cold storage temperature on mass-reared natural enemies.

The storage of parasitoids at low temperature is associated with major fitness costs and the effect on the host often has consequences for the parasitoid. Both the host and the parasitoid are affected by prolonged exposure to cold resulting in sub-lethal or lethal consequences [16]. Cold storage of most of the parasitoid species used in biological control happens under a low (0 °C to 15 °C) temperature, which is suboptimal [1]. This may lead to chilling injury, which induces mortality due to cumulative cold injuries proportional to both the temperature and the duration of exposure [17]. The cold-induced fitness costs [9,18] include reduced reproductive success [19], impaired foraging behavior [20], modified sex ratio [21], higher mortality [22], reduced mobility, flight capacity [4], longevity [23], and fecundity [24]. 

During a fluctuating cold storage regime, the low temperature storage is regularly interrupted by a return to a higher temperature for a short time [9]. In some cases, this will significantly increase the survival with respect to storage at constant, low temperature [2,13,14,15]. Leopold et al. [13] reported that three species of flies, which were transferred to 28 °C for 2–3 h during cold storage, had a significantly increased survival compared to storage at a constant 10 °C. Daily transfer of *Alphitobius diaperinus* stored at 5 °C to a higher temperature 20° C for 2 h allows for the progressive repair of chilling-induced injuries [14]. *Aphidius colemani* emerges earlier when the cold storage of mummies is interrupted by exposing them to 20 °C for 2 h [9]. Fluctuating temperature regimes are also less stressful than constant ones for *Aphidus ervi* [15]. One of the reasons why fluctuating cold storage improves the fitness of emerging insects could be that even a short exposure to higher temperatures allows for the repair of at least some chill injuries [25]. In addition to temperature, the duration of cold exposure is also an important factor for survival. The interaction of these two factors determines the mortality rate [2,26]. However, considering species-specific responses to low temperatures, the possibility of a cold-induced differential mortality is obvious. Hence, cold storage regimes require optimization for individual species to minimize the negative effects on fitness. Many previous studies indicated that survival is affected by the developmental stages exposed to cold storage [27,28]. In Diptera, eggs are more cold-tolerant than adults [27]. The parasitoid *Lysiphlebus fabarum* stored at pupal stage has higher survival than the larval one when stored at 6–8 °C for 1–3 weeks [29]. The higher survival rate of the adult stage over immature stages (eggs, larvae, and pupae) in the eulophid wasp *Tetrastichus brontispae* was reported when stored at 2 °C for 4–48 h [28]. Likewise, cold storage tolerance varies within life stages. Such cold tolerance variation within the pupal stage is reported in several species, including *Trissolcus basalis* and *Trissolcus podisi* [30], *Aphidius rhopalosiphi* [24]; *Encarsia formosa* [31] and *Encarsia sophia* [22]. 

In this study, we investigated the effect of cold storage on *En. sophia* Girault and Dodd (Hymenoptera: Aphelinidae) and *Eretmocerus hayati* Zolnerowich and Rose (Hymenoptera: Aphelinidae), two key parasitoids of the important whitefly pest *Bemisia tabaci* Gennadius (Hemiptera: Aleyrodidae) Middle East-Asia Minor 1 (MEAM1, also called biotype B) and Mediterranean (MED, also called biotype Q) [32,33,34,35]. These two parasitoid wasps reduce *B. tabaci* densities and are recognized as useful biological control agents against *B. tabaci* MEAM1 and MED [34,36,37,38,39,40].

A successful implementation of parasitoid release involves mass-rearing these wasps for release. The parasitoid wasps have to be kept in cold storage to provide sufficient supply during an eventual pest outbreak. However, little is known about the effect of cold storage on their performance, although that information is vital in determining their use in biological control programs. Therefore, this study aimed to determine the effect of fluctuating cold storage regimes on *En. sophia* and *Er. hayati* pupae. The effect of storage was evaluated in terms of fitness parameters, including the emergence rate, time to emergence, adult body size, longevity and parasitization performance. The objective was to identify storage conditions that would delay the emergence of wasps with a minimal impact on their fitness. The results indicated that the storage of old pupal ages at 12 and 10 °C under fluctuating temperatures (transferred every 22 h to 26 ± 1 °C for 2 h) can preserve *En. sophia* and *Er. hayati* for 1 week, respectively, with no or little adverse effect.

## 2. Materials and Methods 

### 2.1. Insects

The whitefly *B. tabaci* MEAM1 used in this experiment was previously maintained in a rearing room for seven years without any exposure to pesticides at Langfang Experiment Station (39°30′ N, 116°36′ E), Institute of Plant Protection, Chinese Academy of Agricultural Sciences (CAAS). The whiteflies were reared under controlled conditions at 26 ± 1 °C, 50 ± 10% relative humidity (RH) and under a photoperiod 14 h:10 h light:dark regime (L:D). Cotton *Gossypium hirsutum* L. cv. Zhong-Mian 8 was used as a host plant for rearing the whitefly. The parasitoids *Er. hayati* and *En. sophia* were reared separately on the whitefly host under the same controlled conditions as the whiteflies. Further details on rearing are available in Kidane et al., [22] and Yang and Wan [34].

### 2.2. Temperature Treatments 

Cold storage experiments were carried out using the pupal stage of *En. sophia and Er. hayati.* To obtain the parasitoid pupae, cotton plants infested with high numbers of 2nd–3rd and 3rd–4th instar nymphs of *B. tabaci* MEAM1 were exposed to *Er. hayati* and *En. sophia,* respectively, for 48 h in a rearing cage 50 cm × 50 cm × 90 cm (width × length × height). Parasitoids at two different pupal ages were used, a young and an older one. For *En. sophia*, these were 10 and 12 days old, both were black colored with the eyes of the latter stage being relatively bulged; for *Er. hayati*, which were 12 and 15 days old, the earlier had no eyes visible and the latter had pink-colored eyes. Appropriately aged pupae were carefully collected from cotton leaves and randomly assigned to the storage temperature treatments. Ten pupae were placed in a vial (1.5 cm diameter and 3 cm long), which was closed with cotton wool. Vials were transferred to 5 different climatic chambers (Saife Instruments, PRX-450D-30, 0–50 ± 1 °C, 50–90% ± 5% RH) which were set at 12, 10, 8, 6 and 4 °C (±1 °C), 60 ± 5% RH and full darkness. In each cold storage treatment, parasitoids were transferred every 22 h to 26 ± 1 °C for 2 h, while in the control treatment, the pupae were kept at a constant 26 ± 1 °C. Two storage periods, 1 and 2 weeks long, were tested. After storage, the pupae were transferred to standard rearing conditions of 26 ± 1 °C, 60 ± 5% RH and 14 h:10 h L:D photoperiod. The fitness parameters, including percentage of emergence, time to emergence, adult body size, longevity and ability to parasitize *B. tabaci* were measured for both species. Each temperature regime treatment had 10 replicates, with each replicate having 10 individuals. A total of 100 pupae were used in each combination of storage temperature, storage time and pupal age.

### 2.3. Parasitoid Fitness after Storage

#### 2.3.1. Emergence Rate and Time

Emergence was checked once a day at the same time (08:30–09:30). The emergence rate was calculated based on the number of individuals that emerged from the total pupae in each vial. The lag time before emergence was calculated from the time when individuals were transferred to standard conditions (26 ± 1 °C) from the cold storage treatment to the day of successful emergence. For the control treatment, since the parasitoids were kept under standard conditions, the emergence time was calculated from pupation to adult emergence. The proportion of emergent adults that emerged over time in every treatment was calculated by dividing the number of adults that emerged each day by the total number of adults that emerged. 

#### 2.3.2. Longevity

The longevity of the adult parasitoids was evaluated by checking the survival daily at 9:30–10:00. For *En. sophia*, we only checked females, since the species are autoparasitoids [37], and these were provided with primary hosts in the test. For *Er. hayati*, both females and males were checked. Newly emerged (<24 h) adults from each treatment were kept individually in small vials (1.5 cm diam., 3-cm long), closed with a moist cotton wool plug. The vials were kept at 26 ± 1 °C, 60 ± 5% RH and 14:10 L:D. A drop of 5% honey solution was provided daily until the wasp died. Twenty-five to 30 individuals were evaluated for most treatments, except the 4 °C and 6 °C temperature treatments, where, due to limited emergence and thus adult availability, 11–25 adults were used. 

#### 2.3.3. Adult Size

The size of the emerged adults was evaluated by measuring the hind tibia (0.1-mm precision). An ocular micrometer mounted on a compound microscope (Olympus, SZX-ILLD2-200) at a magnification of 90× was used. 

#### 2.3.4. Parasitism

To evaluate the parasitism rate, 10 *Er. hayati* females (<24 h old) were randomly selected from each treatment (including the control) and placed individually in a vial with a male for 20 min for mating. No *En. sophia* males emerged from the pupae in any of the treatments; thus, males not exposed to any cold storage treatment were used. After mating, 40 2nd–3rd or 3rd–4th instar nymphs of *B. tabaci* MEAM1 on cotton leaf discs were offered for parasitization to *Er. hayati* and *En. sophia*, respectively. The cotton leaf disc containing the host was placed in a Petri dish with a layer of 1% agar solution, covered by a plastic film. To provide aeration, 30–35 holes were made on the plastic film using an insect pin. The females were removed after 48 h, and the Petri dishes were kept in a climatic chamber at 26 ± 1 °C, 60 ± 5% RH and 14:10 L:D. To maintain the humidity, the vials were kept in a rectangular plastic box (18 cm long × 12 cm wide × 6 cm deep) containing wet filter paper. After 8–12 days, the number of parasitized pupae was recorded. 

### 2.4. Statistical Analysis

Because of the different species and pupal ages evaluated, the effects of the treatment (storage temperature × storage time) on emergence time and rate, longevity, adult body size, and parasitism rates on *B. tabaci* were analyzed separately for *En. sophia* and *Er. hayati*. For both parasitoid species, the effects of the treatment and pupal age on emergence time and rate, longevity, adult body size, and parasitism rates on *B. tabaci* were evaluated using linear regression (Table S1: Best linear regression models selected via Akaike Information Criterion values for *Encarsia sophia* and *Eretmocerus hayati*). Model selection was done accordingly to the Akaike Information Criterion [41]. Model validation was done by assessing the model residuals graphically [42]. Significant differences between treatments were analyzed using the post-hoc test in the R package *lsmeans* [43] using Holm correction for multiple comparisons. For all analyses, we used the R software [44] through R Studio [45].

## 3. Results

### 3.1. Emergence Rate

The emergence rate of *En. sophia* decreased after cold storage treatments (Table 1). For 10-day-old pupae, the emergence rate after cold storage at different temperatures for 1 and 2 weeks was significantly lower than the control (Table S2: Results of post-hoc test of emergence rate) except for the ones stored at 12 and 10 °C, and at 8 °C for one week. For 12-day-old pupae, the emergence rates when stored at 12, 10, 8 and 6 °C for 1 week were not significantly different from the control (Table 1); however, when the pupae were stored longer than 1 week, the emergence rate of pupae decreased significantly (*p* < 0.001 for all comparisons, Table S2). Twelve-day-old pupae, after cold storage at 8 and 6 °C for 1 week, had significantly higher emergence rates than those of 10-day-old ones (post-hoc, *p* = 0.046 and *p* = 0.023, respectively), while storage at 4 °C for 1 week had a significantly lower emergence rate (post-hoc, *p* = 0.023). However, there was no significant difference after two weeks storage at 8, 6 or 4 °C (Table S2).

The emergence rate of *Er. hayati* was reduced at lower temperatures (8, 6 and 4 °C) and longer storage periods compared to the control (Table 1). For 12-day-old pupae, the emergence rate after cold storage for a week at 12 and 10 °C was not significantly different from the control (Table 1). We found no significant difference, either, for 15-day-old pupae stored at 12, 10, 8 and 6 °C for 1 week (Table 1). However, the emergence rate decreased significantly when pupae were stored at 4 °C for one and two weeks (post-hoc, *p* < 0.001 for both). The emergence rate of 15-day-old pupae after cold storage at 12, 10 and 4 °C for 1 week was not different from that of 12-day-old ones (Table S2). However, 15-day-old pupae stored at 8 and 6 °C kept for 1 (*p* = 0.017 and *p* = 0.002, respectively) or at 6 °C for 2 weeks (*p* < 0.001) showed significantly higher emergence rate. The male emergence rate was significantly lower (linear model, *p* < 0.001) than that for females.

### 3.2. Emergence Time

**Figure 1:**
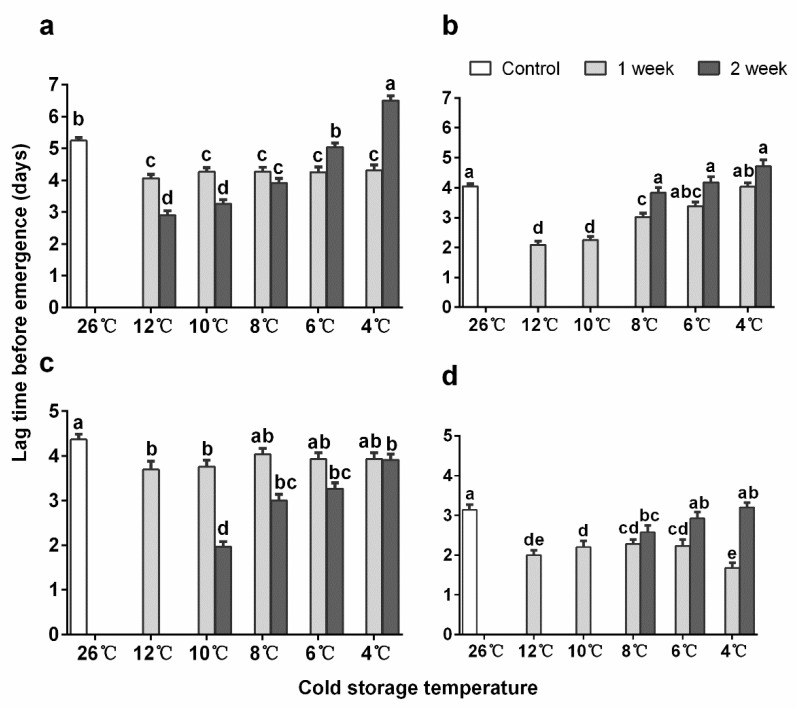
Mean (+ SE) adult emergence time after cold storage of *Encarsia sophia* and *Eretmocerus hayati* pupae in different temperatures. (**a**) The 10-day-old pupal stage and (**b**) 12-day-old pupal stage of *En. sophia*, (**c**) 12-day-old pupal stage and (**d**) 15-day-old pupal stage of *Er. hayati*. Bar heads with different letters in each cluster indicate significant differences in time before emergence among different storage temperatures. (post-hoc test with Holm correction for multiple comparisons on the linear regression with parasitoid emergence time as the response).

The average emergence times of *En. sophia* pupae were significantly shorter for 12-day-old pupae than for 10-day-old pupae (*p* < 0.001). For 10-day-old pupae, the time between the end of the cold treatment to emergence was significantly shorter than in the control, except for the ones stored at 6 and 4 °C for 2 weeks which used similar and longer time to emerge, respectively (Figure 1a, Table S3: Results of post-hoc test of emergence time). For 12-day-old pupae stored for 1 week, the time needed for emergence after storage was significantly reduced when stored at 12, 10, 8 and 6 °C, except for the ones stored at 4 °C, with no significance compared to the control. After 2 weeks of storage, the time to emergence was not significantly different from the control after storage at 8, 6, and 4 °C (Figure 1b). Additionally, most of the adults stored at 4 °C started to emerge on day 1 and 10-day-old pupae stored for 1 week peaked one day earlier, while 10-day-old pupae stored for 2 weeks, as well as 12-day-old pupae stored for 1 week, peaked three days earlier than the control (Figure S1: The proportion of emergence of *Encarcia sophia* adults on different days after cold storages).

For *Er. hayati*, the mean emergence times after all types of cold storage were significantly shorter for 15-day-old pupae than for 12-day-old pupae (*p* < 0.001). For 10-day-old pupae, all treatments shortened the emergence time, except when pupae were stored at 8 °C for one week and at 4 °C for two weeks (Table S3). For 15-day-old pupae, all treatments shortened the emergence time, except when pupae were stored at 6 and 4 °C for two weeks. Moreover, the mean emergence times were significantly shorter for female than male parasitoids (linear model, *p* < 0.001). Most of the adults started to emerge on day 2, and both the old and young pupae stored at 10 and 8 °C for 1 week had a similar emergence as the control (Figure S2: The proportion of emergence of *Eretmocerus hayati* adults on different days after cold storages).

### 3.3. Longevity 

**Figure 2:**
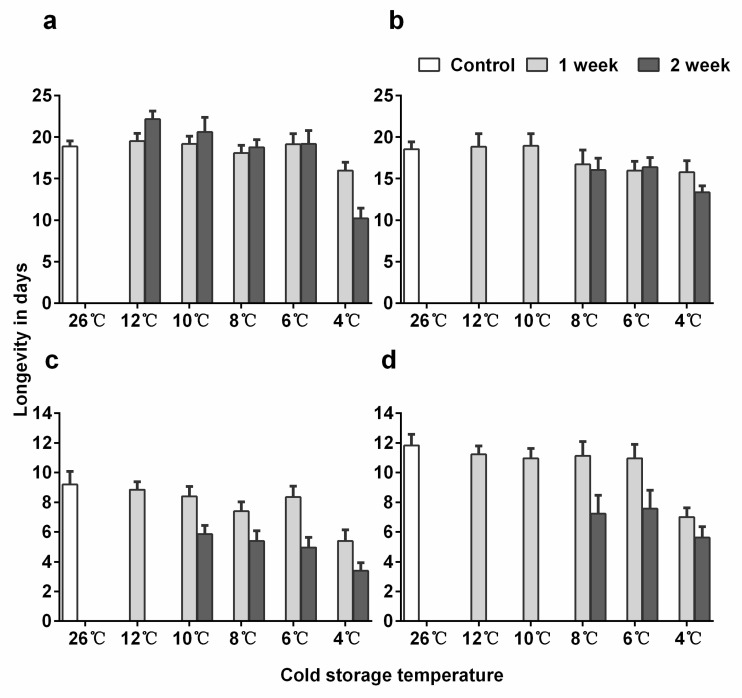
Mean (+ SE) adult longevity of *Encarsia sophia* and *Eretmocerus hayati* females emerged from pupae stored in different cold temperatures. (**a**) The 10-day-old pupal age and (**b**) 12-day-old pupal age of *En. sophia*, (**c**) 12-day-old pupal age and (**d**) 15-day-old pupal age of *Er. hayati*.

In *En. sophia*, adult female longevity was significantly lower for 12-day-old pupae than for 10-day-old ones (*p* = 0.032), but the best model did not include the interaction between pupal age and treatment (Table S4: Results of post-hoc test of adult longevity). Adult female longevity after cold storage was not significantly affected except at 4 °C for two weeks where longevity was significantly reduced (Figure 2a; post-hoc, *p* < 0.001). The longevity of *Er. hayati* adults was significantly higher for female than male parasitoids (Figure S3: Mean adult longevity of *Eretmocerus hayati* males emerged from pupae stored in different cold temperatures), and for 15-day-old pupae than for 12-day-old ones (*p* < 0.001 for both). Cold storage significantly decreased longevity at 10, 8, and 6 °C for two weeks (*p* < 0.001 for all comparisons), and at 4 °C for one and two weeks (*p* < 0.001 for both) (Figure 2c,d).

### 3.4. Adult Body Size

*En. sophia* adults emerging from either pupal age did not show a significant difference in size with respect to the control (Table S5: Results of post-hoc test of body size; Table S6: Effect on hind tibia length (µm) in *Encarsia sophia* after pupal exposure to cold storage at different temperatures and duration). The best model did not include pupal age (Table S1).

The best model explaining *Er. hayati* adult body size did not include the interaction between treatment and pupal age (Table S1). The mean size of *Er. hayati* adults emerged from 12-day-old pupae was significantly higher (*p* < 0.001) than that of adults emerging from 10-day-old pupae. Moreover, male parasitoids body size was significantly larger (*p* < 0.001) than for female parasitoids. When the pupae were kept longer than one week, the mean size decreased significantly (*p* < 0.001 for both, Table S5; Table S7: Effect on hind tibia length (µm) in *Eretmocerus hayati* after pupal exposure to cold storage at different temperatures and duration). 

### 3.5. Parasitization Ability

**Figure 3:**
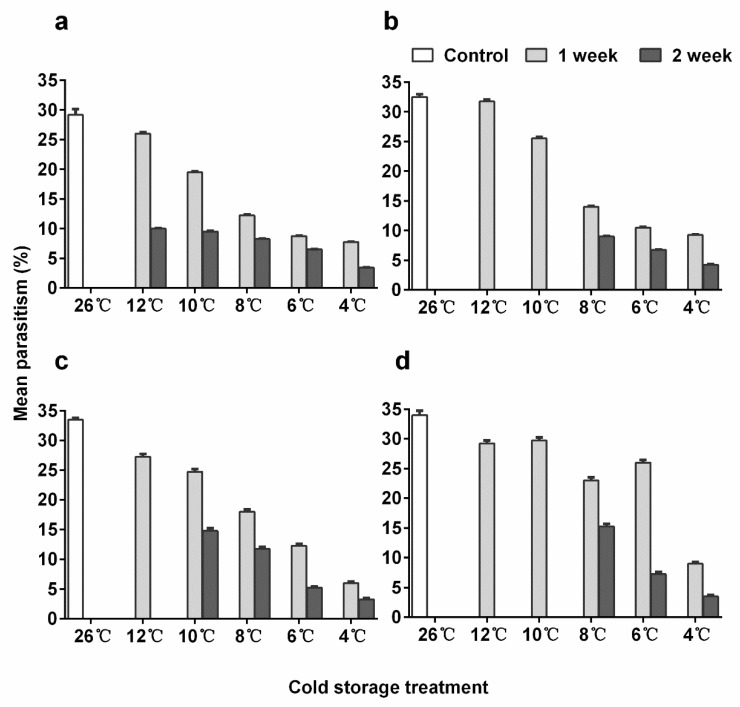
Mean parasitism rate (+ SE) of *Encarsia sophia* and *Eretmocerus hayati* females emerged from pupae stored in different cold temperatures. (**a**) The 10-day-old pupal age and (**b**) 12-day-old pupal age of *En. sophia*, (**c**) 12-day-old pupal age and (**d**) 15-day-old pupal age of *Er. hayati*.

*En. sophia* females emerging from either young or older pupae parasitized significantly fewer hosts in all treatments compared to the control except for 12-day-old pupae kept at 12 °C for 1 week (Figure 3a,b; post-hoc *p* < 0.001 for both 10 and 12-day-old pupae, Table S8: Results of post-hoc test of parasitism rate on *Bemisia tabaci*). The numbers of hosts parasitized decreased as cold storage temperatures decreased and storage time increased. The parasitism by females emerged from 12-day-old pupae stored at 12 °C and 10 °C for 1 week was significantly higher than the ones that emerged from 10-day-old pupae kept under the same conditions (post-hoc, *p* < 0.001 for both, Table S8). The parasitism after 12 old pupae were stored at 10 °C for 1 week was 25.5%, 7% lower than in the control.

The parasitization ability in *Er. hayati* was significantly higher (linear regression, *p* < 0.001) in adults emerging from 15-day-old pupae than from 10-day-old ones. The best model did not include the interaction between pupal age and treatment, and all cold treatments reduced the parasitization ability in females (Figure 3c,d; post-hoc, *p* < 0.001 for all comparisons, Table S8). This negative effect was stronger with a decreasing storage temperature and increasing storage time. The parasitism after 15-day-old pupae were stored at 10 °C for 1 week was 29.8%, 4.2% less than the control.

## 4. Discussion

The combination of exposure time and temperature determines the severity of cold-induced injury and a decrease in temperature and/or an increase in exposure time can result in cumulative and irreversible chilling injuries [1]. Exposure to prolonged cold storage has a negative effect on the emergence of parasitic wasps [4,23]. The level of accumulated injury increases as the duration of exposure increases [25] and chilling injury accumulates [1] and eventually becomes lethal. The results of this study are in accordance with this general theory showing a significant decrease in emergence rate of both studied parasitoids with increasing exposure. 

Younger pupae of parasitoids are more sensitive to cold storage, and have reduced emergence rates. For example, *T.basalis* and *T. podisi* pupae are less cold tolerant when stored as young compared to late ones [30]. Cold storage of earlier developmental stages drastically reduces the emergence in *A. ervi* [46]. In the present study, the younger pupae of both *En. sophia* and *Er. hayati* were less cold tolerant than the older ones, reflected by their lower parasitism ability.

Exposure to a prolonged constant low temperature with frequent transfers to optimal temperature can reduce the cold-induced damage [9,29]. The use of fluctuating temperature improved the performance of several insect species that are strongly affected under constant temperature, such as the lesser mealworm *A. diaperinus* [14], the heteropteran *Pyrrhocoris apterus* [25], and the solitary bee *Megachile rotundata* [12]. Colinet et al. [9] reported a higher survival of *A. colemani* when pupae were exposed to daily warming at 20 °C than those exposed to a constant low temperature at 4 °C. In the present study, a remarkable improvement in the emergence of the two parasitoids was observed when the pupae were transferred to their optimal temperature (26 °C) for 2 h daily. The emergence rate of both early and late pupal stages of *En. sophia* is only 41–43% when stored at 12 °C for 2 weeks [22], while, in the present study, the emergence rate increased to 80–84% when the same cold storage conditions included a transfer to their optimal temperature for 2 h every day. Likewise, young (12-day-old) *Er. hayati* pupae stored at a constant 12 °C for 1 or 2 weeks had an emergence rate of 35% and 0%, respectively, and 15-day-old pupae showed 52% and 27% emergence rates, respectively [47]. However, in the present study, a daily short exposure to higher temperatures increased the emergence rate to 88% from both younger and older pupae. These results confirm the positive impact of the periodical transfer to optimal temperature on the emergence of the adults from cold-exposed pupae. Periodic exposure to an optimal temperature reduces the amount of accumulated injuries and allows for the reactivation of metabolic processes necessary for development [10,25]. 

Cold storage may affect the genders differentially. There is a shift towards higher proportions of males after the cold storage of the pupae, as found in *Telenomus busseolae* [48], *Aphidius rhopalosiphi* [49] and *Gonatocerus ashmeadi* [5]. However, we found that the sex ratio of *Er. hayati* was not affected by cold storage (Table S9). Hence, there was no differential pupal mortality depending on gender due to cold storage. However, the adult longevity, emergence rate and body size of *Er. hayati* females were higher than those of males, while the emergence time was shorter. Similar results for *Er. corni* were reported by Lopez and Botto [50].

The time required for adult emergence after cold storage is expected to decrease with the increasing duration of cold exposure [9]. Contrary to this, the emergence time in *En. sophia* after storage at a constant <12 °C temperature increases with its duration [22], suggesting that, under those conditions, development is arrested. Here, we found that the lag time before emergence decreased with the longer cold storage when the pupae of *En. sophia* were kept at 12 and 10 °C with periodic exposure to a higher temperature. No such decrease was observed when the pupae were stored at <8 °C. For *Er. hayati,* the lag time before emergence significantly decreased with increasing storage duration when younger pupae were stored. This indicated that the 2 h daily transfer to an optimal temperature allowed them to continue developing. This variation between the two parasitoid species emphasizes the difficulty in generalizing about the relationship between emergence time and cold storage duration. A daily 2 h transfer of cold-stored *A. colemani* and *A. ervi* pupae to an optimal temperature decreases their emergence time [9,15], while a delay in emergence time is observed in other insect species after cold storage, such as *Trissolcus semistriatus* [51], *G.ashmeadi* [52] and in the fly *Scatophaga stercoraria* [53].

The longevity of *En. sophia* under a constant low temperature for 2 weeks is severely reduced [22]. In the present study, the longevity of *En. sophia* as well as of *Er. hayati* was extended when the cold storage was interrupted by periodic, short exposure to an optimum temperature, similar to *Trichogramma galloi* [54], *A. colemani* [9] and *A. rhopalosiphi* [24]. Exposure to constant low temperature severely injured *Er. hayati* males that were weak and the majority of them died in the first few hours after emergence [47], while this can also be “cured” by periodic exposure to higher temperatures.

Exposure to a constant suboptimal temperature usually negatively affects the parasitization ability of the emerging parasitoids [16], which was also demonstrated for *En. sophia* [22]. Under our experimental conditions, the parasitism by *En. sophia* was increased, suggesting that fluctuating temperature storage regime benefits the parasitoid. Likewise, emerged *Er. hayati* adults were viable and parasitism by females emerging from older pupae stored at 10 °C for one week was not significantly reduced. This substantial reduction in the negative effect of cold storage is probably due to the transfer of the pupae to the optimum temperature for a short period. Mahi et al. [29] showed that the parasitism by *L. fabarum* after being stored at 6 °C for 2 weeks was similar to the control under a fluctuating storage temperature. The fecundity of *A. colemani* was also improved by such storage conditions [18].

## 5. Conclusions

The cold storage of *En. sophia* and *Er. hayati* pupae below 10 °C strongly influenced the fitness of the emerging adults. A short daily exposure to a higher temperature during cold storage clearly improves the performance of the parasitoid when they are stored at their late pupal stage. In particular, in the case of *Er. hayati*, in addition to a higher emergence and increased longevity, fluctuating cold storage preserves the viability of the males, which is not the case under constant cold storage temperatures. Based on the results of this study, it is concluded that the late pupal stages of both *En. sophia* and *Er. hayati* can be stored for one week at 12 °C without detrimental effects on their fitness and at 10 °C with a moderate loss of fitness (parasitism) under short daily exposure to higher temperatures.

## Figures and Tables

**Table 1:** Emergence rates of *Encarsia sophia* and *Eretmocerus hayati* after storage at different pupal ages exposed to different temperatures and duration. Data are means ± standard error (SE).

Storage Treatment	Emergency Rate (%)			
	*Encarsia sophia*		*Eretmocerus hayati*	
	10-Day-Old Pupae	12-Day-Old Pupae	12-Day-Old Pupae	15-Day-Old Pupae
Control (26 °C)	86.0 ± 0.3 aA ^1^	87.0 ± 0.3 aA	90.0 ± 0.2 aA	85.0 ± 0.3 aA
12 °C/1 week	81.0 ± 0.3 abA	84.0 ± 0.4 aA	88.0 ± 0.3 aA	88.0 ± 0.2 aA
12 °C/2 weeks	80.0 ± 0.3 ab	⊗ ^2^	⊗	⊗
10 °C/1 week	75.0 ± 0.3 abA	85.0 ± 0.3 aA	87.0 ± 0.3 aA	86.0 ± 0.3 aA
10 °C/2 weeks	75.0 ± 0.3 ab	⊗	77.0 ± 0.3 ab	⊗
8 °C/1 week	72.0 ± 0.3 abcB	88.0 ± 0.3 aA	62.0 ± 0.4 abcB	86.0 ± 0.3 aA
8 °C/2 weeks	57.0 ± 0.4 cA	53.0 ± 0.3 bA	59.0 ± 0.3 bcA	75.0 ± 0.3 aA
6 °C/1 week	67.0 ± 0.4 bcB	84.0 ± 0.2 aA	56.0 ± 0.3 bcB	84.0 ± 0.3 aA
6 °C/2 weeks	49.0 ± 0.3 cA	41.0 ± 0.6 bA	45.0 ± 0.4 cdB	77.0 ± 0.3 aA
4 °C/1 week	56.0 ± 0.5 cA	39.0 ± 0.3 bB	43.0 ± 0.3 cdA	40.0 ± 0.3 bA
4 °C/2 weeks	18.0 ± 0.2 dA	14.0 ± 0.3 cA	22.0 ± 0.1 dA	30.0 ± 0.3 bA

^1^ Means followed by different lower case letters within the same column were significantly different; means followed by different upper case letter within the same row of each species were significant different (post-hoc test with Holm correction for multiple comparisons on the linear regression with parasitoid emergence rate as the response). ^2^ ⊗ discarded because pupae started to emerge during cold storage.

## Supplementary Materials

The following are available online at https://www.mdpi.com/2075-4450/11/7/428/s1. Figure S1: The proportion of emergence (Mean + SE) of *Encarcia sophia* adults on different days after cold storages, Figure S2: The proportion of emergence (Mean + SE) of *Eretmocerus hayati* adults on different days after cold storages, Figure S3: Mean (+ SE) adult longevity of *Eretmocerus hayati* males emerged from pupae stored in different cold temperatures, Table S1: Best linear regression models selected via Akaike Information Criterion values for *Encarsia sophia* and *Eretmocerus hayati*, Table S2: Results of the post-hoc test with Holm correction for multiple comparisons on the linear regression with parasitoid emergence rate as the response. The post-hoc was performed through the R package lsmeans. Significant differences are given in bold. Table S3: Results of the post-hoc test with Holm correction for multiple comparisons on the linear regression with parasitoid emergence time as the response. The post-hoc was performed through the R package lsmeans. Significant differences are given in bold. Table S4: Results of the post-hoc test with Holm correction for multiple comparisons on the linear regression with adult parasitoid longevity as the response. The post-hoc was performed through the R package lsmeans. Significant differences are given in bold. Table S5: Results of the post-hoc test with Holm correction for multiple comparisons on the linear regression with adult body size as the response. The post-hoc was performed through the R package lsmeans. Significant differences are given in bold. Table S6: Effect on hind tibia length (µm) in *Encarsia sophia* after pupal exposure to cold storage at different temperatures and duration, Table S7: Effect on hind tibia length (µm) in *Eretmocerus hayati* after pupal exposure to cold storage at different temperatures and duration, Table S8: Table S8. Results of the post-hoc test with Holm correction for multiple comparisons on the linear regression with parasitism rate on *Bemisia tabaci* as the response. The post-hoc was performed through the R package lsmeans. Significant differences are given in bold. Table S9: Effect of cold storage on sex ratio of emerged *Eretmocerus hayati* after the pupae exposed to different temperatures and duration.
