# Supplementary material for: Cold Storage Effects on Fitness of the Whitefly Parasitoids Encarsia sophia and Eretmocerus hayati
Source: Insects. 2020 Jul 9;11(7):428. doi: 10.3390/insects11070428 (PMC7412127; doi:10.3390/insects11070428)
Supplement: Supplementary file 1 [file insects-11-00428-s001.pdf]

Supplementary Material for

Cold storage effects on fitness of the whitefly parasitoids *Encarsia sophia* and *Eretmocerus hayati*

The proportion of emergence adults that emerged over time

The emergence period of *En. sophia* from the control treatment (26°C) lasted 4 days; for 10 and 12-day-old pupae, adults started to emerge on day 3 and 2, reached the maximum on the sixth and fourth days, respectively (Figure S1). After 1 week cold storage, adults began to emerge on day 1 and the emergence period lasted 4–6 days for both 10 and 12 day old pupae. Most adults emerged on day 5 in almost all cold storage treatments (except for 8°C in which emergence peaked on day 4) and was one day earlier than the control for 10-day-old pupae (Figure S1a). For 12-day-old pupae, most adults emerged on day 1 in the 12 and 10°C treatments, on day 3 for the 8°C treatment, which was 3 and 1 days earlier than that in the control, respectively (Figure S1c). For 2 weeks cold storage, adult emergence lasted 3–5 days and 4–5 days for 10 and 12-day-old pupae, respectively. For 10-day-old pupae, adults began to emerge on day 1 and most of them emerged on day 3, and days 2–3 when stored at 12 and 10°C, respectively, which is around 3 days earlier than that of control (Figure S1b). For 12 day old pupae, adults began to emerge and reached peak emergence on days 4–5 in 8, 6 and 4°C treatments, which was similar to the control (Figure S1d).

For *Er. hayati*, in the control treatment (26°C), the emergence period of the parasitoids last 3–5 days; for 12 and 15-day-old pupae, adults started to emerge on day 3 and 2, reached the maximum on the fourth and third day (Figure S2). After one week in cold storage, adults began to emerge on day 2 and 1, emerged over a period of 2–6 and 1–4 days, for younger and old old pupae, respectively; with a peak on day 3 or 4 (Figure S2a, c). After two weeks of cold storage, adults emerged over 3–4 and 2–3 days for 12 and 15-day-old pupae, respectively (Figure S2b, d).

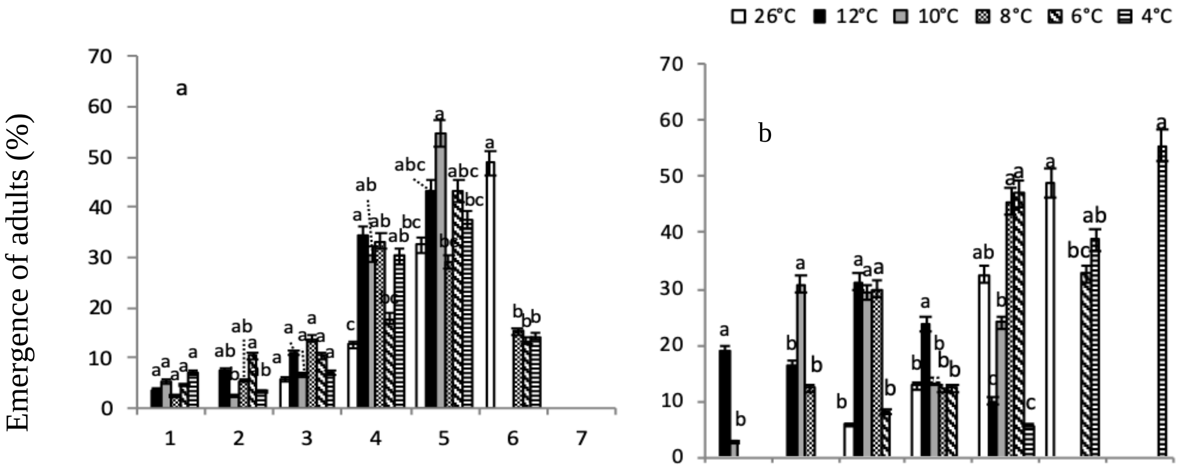

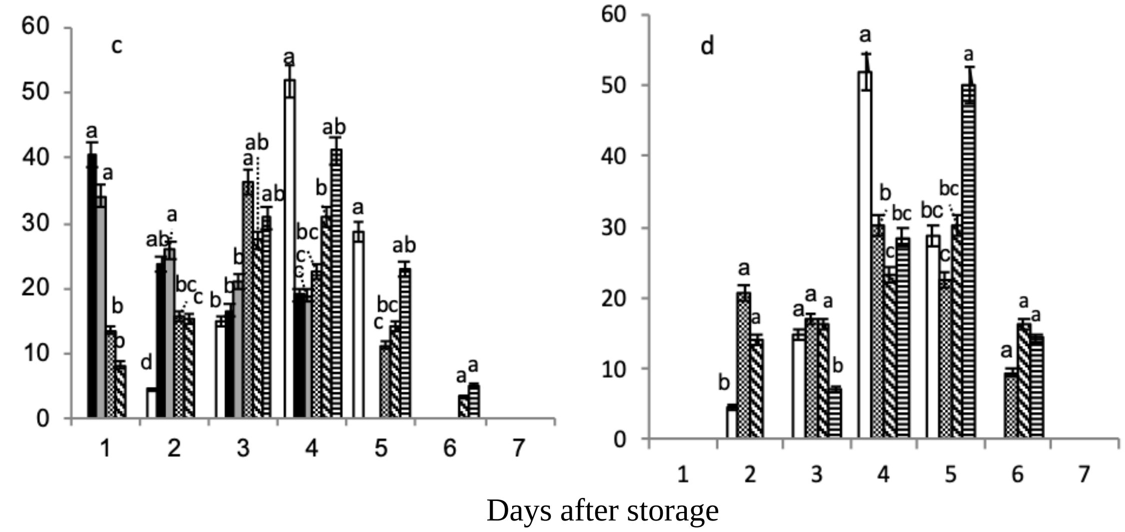

Figure 1. The proportion of emergence (Mean + SE) of *Encarcia sophia* adults on different days after cold storages: **(a)** 10-day-old pupae after 1 week storage period, **(b)** 10-day-old pupae after 2 week storage period, **(c)** 12-day-old pupae after 1 week storage period and **(d)** 12-day-old pupae after 2 week storage period. Bar heads with different letters in each cluster indicate significantly differences in proportion of emergence among different storage temperatures.

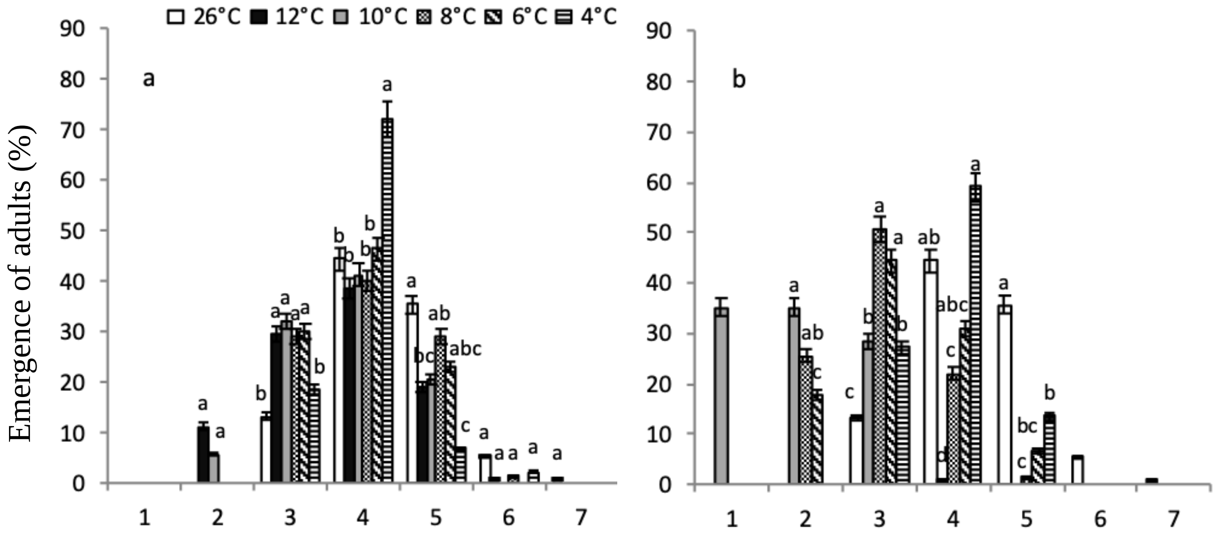

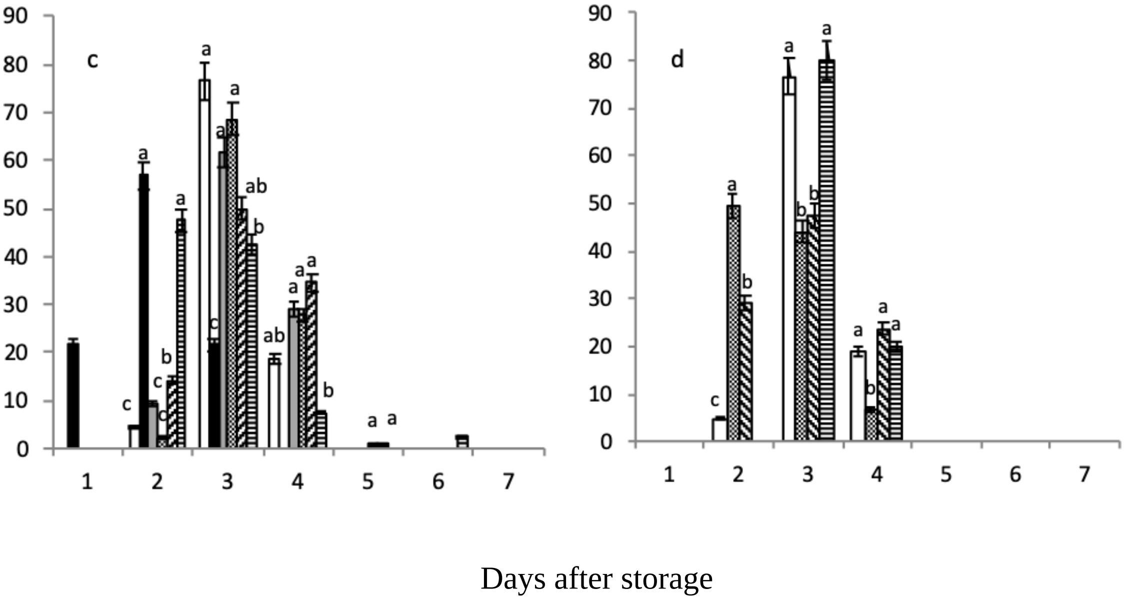

Figure S2. The proportion of emergence (Mean + SE) of *Eretmocerus hayati* adults on different days after cold storages: (a) 12-day-old pupae after 1 week storage period, (b) 12-day-old pupae after 2 week storage period, (c) 15-day-old pupae after 1 week storage period and (d) 15- day-old pupae after 2 week storage period. Bar heads with different letters in each cluster indicate significantly differences in proportion of emergence among different storage temperatures.

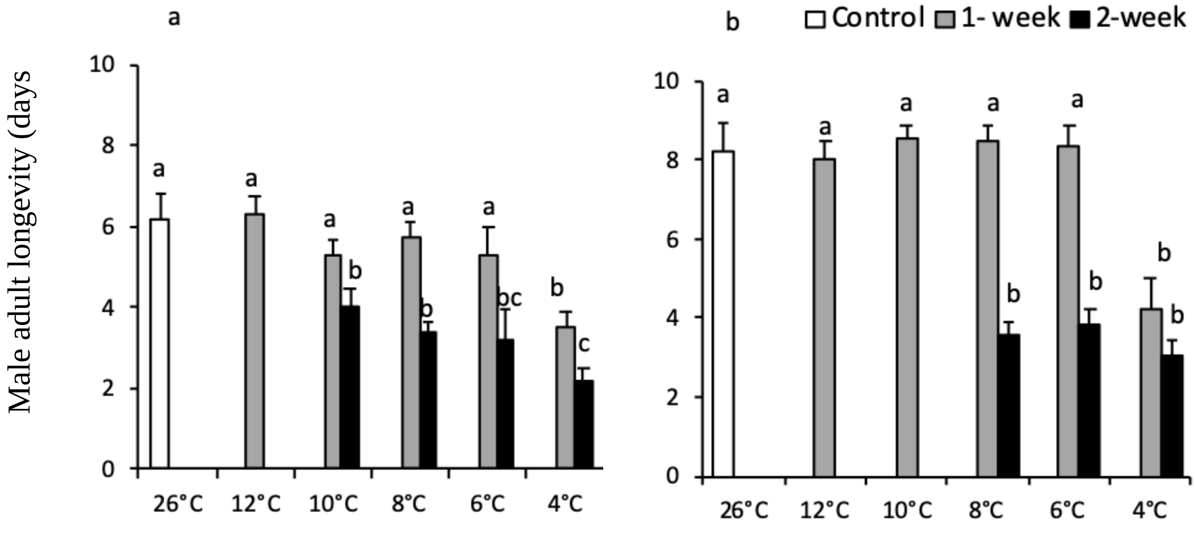

Figure 3. Mean (+ SE) adult longevity of *Eretmocerus hayati* males emerged from pupae stored in different cold temperatures. **(a)** male emerged from 12-day-old pupae, **(b)** male emerged from 15-day-old pupae. Bar heads with different letters indicate significant differences.

Table 1. Best linear regression models selected via Akaike Information Criterion values for *Encarsia sophia* and *Eretmocerus hayati*.

Species	Response	Best model	Adj. R2 (%)	Significance of the model
Encarsia sophia	Emergence time	Treatment * Pupal age	39.0	*P* < 0.001
	Emergence rate	Treatment * Pupal age	81.0	*P* < 0.001
	Longevity	Treatment + Pupal age	8.8	*P* < 0.001
	Body size	Treatment	1.9	*P* = 0.068
	Parasitism rate	Treatment + Pupal age	92.4	*P* < 0.001
Eretmocerus hayati	Emergence time	Treatment * Pupal age + Sex	55.9	*P* < 0.001
	Emergence rate	Treatment * Pupal age + Sex	51.1	*P* < 0.001
	Longevity	Treatment + Pupal age + Sex	38.5	*P* < 0.001
	Body size	Treatment + Pupal age + Sex	13.8	*P* < 0.001
	Parasitism rate	Treatment + Pupal age	88.6	*P* < 0.001

Table 2. Results of the post hoc test with Holm correction for multiple comparisons on the linear regression with parasitoid emergence rate as the response. The post hoc was performed through the R package lsmeans. Significant differences are given in bold.

Species	Contrast	Estimate	SE	df	t.ratio	Significance
Encarsia sophia	Control.10-day-old - 12C_1w.10-day-old	0.05	0.048	180	1.044	1
	Control.10-day-old - 12C_2w.10-day-old	0.06	0.048	180	1.253	1
	Control.10-day-old - 10C_1w.10-day-old	0.11	0.048	180	2.298	0.841
	Control.10-day-old - 10C_2w.10-day-old	0.11	0.048	180	2.298	0.841
	Control.10-day-old - 8C_1w.10-day-old	0.14	0.048	180	2.924	0.164
	Control.10-day-old - 8C_2w.10-day-old	0.29	0.048	180	6.057	**P < 0.001**
	Control.10-day-old - 6C_1w.10-day-old	0.19	0.048	180	3.968	**0.005**
	Control.10-day-old - 6C_2w.10-day-old	0.37	0.048	180	7.728	**P < 0.001**
	Control.10-day-old - 4C_1w.10-day-old	0.3	0.048	180	6.266	**P < 0.001**
	Control.10-day-old - 4C_2w.10-day-old	0.68	0.048	180	14.203	**P < 0.001**
	Control.10-day-old - Control.12-day-old	-0.01	0.048	180	-0.209	1
	12C_1w.10-day-old - 12C_2w.10-day-old	0.01	0.048	180	0.209	1
	12C_1w.10-day-old - 10C_1w.10-day-old	0.06	0.048	180	1.253	1
	12C_1w.10-day-old - 10C_2w.10-day-old	0.06	0.048	180	1.253	1
	12C_1w.10-day-old - 8C_1w.10-day-old	0.09	0.048	180	1.88	1
	12C_1w.10-day-old - 8C_2w.10-day-old	0.24	0.048	180	5.013	**P < 0.001**
	12C_1w.10-day-old - 6C_1w.10-day-old	0.14	0.048	180	2.924	0.164
	12C_1w.10-day-old - 6C_2w.10-day-old	0.32	0.048	180	6.684	**P < 0.001**
	12C_1w.10-day-old - 4C_1w.10-day-old	0.25	0.048	180	5.222	**P < 0.001**
	12C_1w.10-day-old - 4C_2w.10-day-old	0.63	0.048	180	13.159	**P < 0.001**
	12C_1w.10-day-old - 12C_1w.12-day-old	-0.03	0.048	180	-0.627	1
	12C_2w.10-day-old - 10C_1w.10-day-old	0.05	0.048	180	1.044	1
	12C_2w.10-day-old - 10C_2w.10-day-old	0.05	0.048	180	1.044	1
	12C_2w.10-day-old - 8C_1w.10-day-old	0.08	0.048	180	1.671	1
	12C_2w.10-day-old - 8C_2w.10-day-old	0.23	0.048	180	4.804	**P < 0.001**
	12C_2w.10-day-old - 6C_1w.10-day-old	0.13	0.048	180	2.715	0.283
	12C_2w.10-day-old - 6C_2w.10-day-old	0.31	0.048	180	6.475	**P < 0.001**
	12C_2w.10-day-old - 4C_1w.10-day-old	0.24	0.048	180	5.013	**P < 0.001**
	12C_2w.10-day-old - 4C_2w.10-day-old	0.62	0.048	180	12.95	**P < 0.001**
	10C_1w.10-day-old - 10C_2w.10-day-old	0	0.048	180	0	1
	10C_1w.10-day-old - 8C_1w.10-day-old	0.03	0.048	180	0.627	1
**Species**	**Contrast**	**Estimate**	**SE**	**df**	**t.ratio**	**Significance**
	10C_1w.10-day-old - 8C_2w.10-day-old	0.18	0.048	180	3.76	**0.011**

	10C_1w.10-day-old - 6C_1w.10-day-old	0.08	0.048	180	1.671	1
	10C_1w.10-day-old - 6C_2w.10-day-old	0.26	0.048	180	5.431	**P < 0.001**
	10C_1w.10-day-old - 4C_1w.10-day-old	0.19	0.048	180	3.968	**0.005**
	10C_1w.10-day-old - 4C_2w.10-day-old	0.57	0.048	180	11.905	**P < 0.001**
	10C_1w.10-day-old - 10C_1w.12-day-old	-0.1	0.048	180	-2.089	1
	8C_1w.10-day-old - 8C_2w.10-day-old	0.15	0.048	180	3.133	0.087
	8C_1w.10-day-old - 6C_1w.10-day-old	0.05	0.048	180	1.044	1
	8C_1w.10-day-old - 6C_2w.10-day-old	0.23	0.048	180	4.804	**P < 0.001**
	8C_1w.10-day-old - 4C_1w.10-day-old	0.16	0.048	180	3.342	**0.046**
	8C_1w.10-day-old - 4C_2w.10-day-old	0.54	0.048	180	11.279	**P < 0.001**
	8C_1w.10-day-old - 8C_1w.12-day-old	-0.16	0.048	180	-3.342	**0.046**
	8C_2w.10-day-old - 6C_1w.10-day-old	-0.1	0.048	180	-2.089	1
	8C_2w.10-day-old - 6C_2w.10-day-old	0.08	0.048	180	1.671	1
	8C_2w.10-day-old - 4C_1w.10-day-old	0.01	0.048	180	0.209	1
	8C_2w.10-day-old - 4C_2w.10-day-old	0.39	0.048	180	8.146	**P < 0.001**
	8C_2w.10-day-old - 8C_2w.12-day-old	0.04	0.048	180	0.835	1
	6C_1w.10-day-old - 4C_1w.10-day-old	0.11	0.048	180	2.298	0.841
	6C_1w.10-day-old - 4C_2w.10-day-old	0.49	0.048	180	10.235	**P < 0.001**
	6C_1w.10-day-old - 6C_1w.12-day-old	-0.17	0.048	180	-3.551	**0.023**
	6C_2w.10-day-old - 4C_1w.10-day-old	-0.07	0.048	180	-1.462	1
	6C_2w.10-day-old - 4C_2w.10-day-old	0.31	0.048	180	6.475	**P < 0.001**
	6C_2w.10-day-old - 6C_2w.12-day-old	0.08	0.048	180	1.671	1
	4C_1w.10-day-old - 4C_2w.10-day-old	0.38	0.048	180	7.937	**P < 0.001**
	4C_1w.10-day-old - 4C_1w.12-day-old	0.17	0.048	180	3.551	**0.023**
	4C_2w.10-day-old - 4C_2w.12-day-old	0.04	0.048	180	0.835	1
	Control.12-day-old - 12C_1w.12-day-old	0.03	0.048	180	0.627	1
	Control.12-day-old - 10C_1w.12-day-old	0.02	0.048	180	0.418	1
	Control.12-day-old - 8C_1w.12-day-old	-0.01	0.048	180	-0.209	1
	Control.12-day-old - 8C_2w.12-day-old	0.34	0.048	180	7.102	**P < 0.001**
	Control.12-day-old - 6C_1w.12-day-old	0.03	0.048	180	0.627	**1**
	Control.12-day-old - 6C_2w.12-day-old	0.46	0.048	180	9.608	**P < 0.001**
	Control.12-day-old - 4C_1w.12-day-old	0.48	0.048	180	10.026	**P < 0.001**
	Control.12-day-old - 4C_2w.12-day-old	0.73	0.048	180	15.247	**P < 0.001**
	12C_1w.12-day-old - 10C_1w.12-day-old	-0.01	0.048	180	-0.209	**1**
**Species**	**Contrast**	**Estimate**	**SE**	**df**	**t.ratio**	**Significance**
	12C_1w.12-day-old - 8C_1w.12-day-old	-0.04	0.048	180	-0.835	**1**
	12C_1w.12-day-old - 8C_2w.12-day-old	0.31	0.048	180	6.475	**P < 0.001**

	12C_1w.12-day-old - 6C_1w.12-day-old	0	0.048	180	0	**1**
	12C_1w.12-day-old - 6C_2w.12-day-old	0.43	0.048	180	8.981	**P < 0.001**
	12C_1w.12-day-old - 4C_1w.12-day-old	0.45	0.048	180	9.399	**P < 0.001**
	12C_1w.12-day-old - 4C_2w.12-day-old	0.7	0.048	180	14.621	**P < 0.001**
	10C_1w.12-day-old - 8C_1w.12-day-old	-0.03	0.048	180	-0.627	**1**
	10C_1w.12-day-old - 8C_2w.12-day-old	0.32	0.048	180	6.684	**P < 0.001**
	10C_1w.12-day-old - 6C_1w.12-day-old	0.01	0.048	180	0.209	**1**
	10C_1w.12-day-old - 6C_2w.12-day-old	0.44	0.048	180	9.19	**P < 0.001**
	10C_1w.12-day-old - 4C_1w.12-day-old	0.46	0.048	180	9.608	**P < 0.001**
	10C_1w.12-day-old - 4C_2w.12-day-old	0.71	0.048	180	14.83	**P < 0.001**
	8C_1w.12-day-old - 8C_2w.12-day-old	0.35	0.048	180	7.31	**P < 0.001**
	8C_1w.12-day-old - 6C_1w.12-day-old	0.04	0.048	180	0.835	**1**
	8C_1w.12-day-old - 6C_2w.12-day-old	0.47	0.048	180	9.817	**P < 0.001**
	8C_1w.12-day-old - 4C_1w.12-day-old	0.49	0.048	180	10.235	**P < 0.001**
	8C_1w.12-day-old - 4C_2w.12-day-old	0.74	0.048	180	15.456	**P < 0.001**
	8C_2w.12-day-old - 6C_1w.12-day-old	-0.31	0.048	180	-6.475	**P < 0.001**
	8C_2w.12-day-old - 6C_2w.12-day-old	0.12	0.048	180	2.506	**0.497**
	8C_2w.12-day-old - 4C_1w.12-day-old	0.14	0.048	180	2.924	**0.164**
	8C_2w.12-day-old - 4C_2w.12-day-old	0.39	0.048	180	8.146	**P < 0.001**
	6C_1w.12-day-old - 6C_2w.12-day-old	0.43	0.048	180	8.981	**P < 0.001**
	6C_1w.12-day-old - 4C_1w.12-day-old	0.45	0.048	180	9.399	**P < 0.001**
	6C_1w.12-day-old - 4C_2w.12-day-old	0.7	0.048	180	14.621	**P < 0.001**
	6C_2w.12-day-old - 4C_1w.12-day-old	0.02	0.048	180	0.418	**1**
	6C_2w.12-day-old - 4C_2w.12-day-old	0.27	0.048	180	5.639	**P < 0.001**
	4C_1w.12-day-old - 4C_2w.12-day-old	0.25	0.048	180	5.222	**P < 0.001**
Eretmocerus hayati	Control.12-day-old - 12C_1w.12-day-old	0.02	0.066	360	0.301	**1**
	Control.12-day-old - 10C_1w.12-day-old	0.03	0.066	360	0.451	**1**
	Control.12-day-old - 10C_2w.12-day-old	0.13	0.066	360	1.955	**1**
	Control.12-day-old - 8C_1w.12-day-old	0.28	0.066	360	4.211	**0.002**
	Control.12-day-old - 8C_2w.12-day-old	0.31	0.066	360	4.663	**P < 0.001**
	Control.12-day-old - 6C_1w.12-day-old	0.34	0.066	360	5.114	**P < 0.001**
	Control.12-day-old - 6C_2w.12-day-old	0.45	0.066	360	6.768	**P < 0.001**
	Control.12-day-old - 4C_1w.12-day-old	0.47	0.066	360	7.069	**P < 0.001**

**Species**	**Contrast**	**Estimate**	**SE**	**df**	**t.ratio**	**Significance**
	Control.12-day-old - 4C_2w.12-day-old	0.68	0.066	360	10.228	**P < 0.001**
	Control.12-day-old - Control.15-day-old	0.05	0.066	360	0.752	**1**
	12C_1w.12-day-old - 10C_1w.12-day-old	0.01	0.066	360	0.15	**1**

	12C_1w.12-day-old - 10C_2w.12-day-old	0.11	0.066	360	1.654	**1**
	12C_1w.12-day-old - 8C_1w.12-day-old	0.26	0.066	360	3.911	**0.006**
	12C_1w.12-day-old - 8C_2w.12-day-old	0.29	0.066	360	4.362	**0.001**
	12C_1w.12-day-old - 6C_1w.12-day-old	0.32	0.066	360	4.813	**P < 0.001**
	12C_1w.12-day-old - 6C_2w.12-day-old	0.43	0.066	360	6.468	**P < 0.001**
	12C_1w.12-day-old - 4C_1w.12-day-old	0.45	0.066	360	6.768	**P < 0.001**
	12C_1w.12-day-old - 4C_2w.12-day-old	0.66	0.066	360	9.927	**P < 0.001**
	12C_1w.12-day-old - 12C_1w.15-day-old	0	0.066	360	0	**1**
	10C_1w.12-day-old - 10C_2w.12-day-old	0.1	0.066	360	1.504	**1**
	10C_1w.12-day-old - 8C_1w.12-day-old	0.25	0.066	360	3.76	**0.01**
	10C_1w.12-day-old - 8C_2w.12-day-old	0.28	0.066	360	4.211	**0.002**
	10C_1w.12-day-old - 6C_1w.12-day-old	0.31	0.066	360	4.663	**P < 0.001**
	10C_1w.12-day-old - 6C_2w.12-day-old	0.42	0.066	360	6.317	**P < 0.001**
	10C_1w.12-day-old - 4C_1w.12-day-old	0.44	0.066	360	6.618	**P < 0.001**
	10C_1w.12-day-old - 4C_2w.12-day-old	0.65	0.066	360	9.777	**P < 0.001**
	10C_1w.12-day-old - 10C_1w.15-day-old	0.01	0.066	360	0.15	**1**
	10C_2w.12-day-old - 8C_1w.12-day-old	0.15	0.066	360	2.256	**1**
	10C_2w.12-day-old - 8C_2w.12-day-old	0.18	0.066	360	2.707	**0.32**
	10C_2w.12-day-old - 6C_1w.12-day-old	0.21	0.066	360	3.159	**0.083**
	10C_2w.12-day-old - 6C_2w.12-day-old	0.32	0.066	360	4.813	**P < 0.001**
	10C_2w.12-day-old - 4C_1w.12-day-old	0.34	0.066	360	5.114	**P < 0.001**
	10C_2w.12-day-old - 4C_2w.12-day-old	0.55	0.066	360	8.272	**P < 0.001**
	8C_1w.12-day-old - 8C_2w.12-day-old	0.03	0.066	360	0.451	**1**
	8C_1w.12-day-old - 6C_1w.12-day-old	0.06	0.066	360	0.902	**1**
	8C_1w.12-day-old - 6C_2w.12-day-old	0.17	0.066	360	2.557	**0.483**
	8C_1w.12-day-old - 4C_1w.12-day-old	0.19	0.066	360	2.858	**0.208**
	8C_1w.12-day-old - 4C_2w.12-day-old	0.4	0.066	360	6.016	**P < 0.001**
	8C_1w.12-day-old - 8C_1w.15-day-old	-0.24	0.066	360	-3.61	**0.017**
	8C_2w.12-day-old - 6C_1w.12-day-old	0.03	0.066	360	0.451	**1**
	8C_2w.12-day-old - 6C_2w.12-day-old	0.14	0.066	360	2.106	**1**
	8C_2w.12-day-old - 4C_1w.12-day-old	0.16	0.066	360	2.407	**0.714**
	8C_2w.12-day-old - 4C_2w.12-day-old	0.37	0.066	360	5.565	**P < 0.001**

Species	Contrast	Estimate	SE	df	t.ratio	Significance
	8C_2w.12-day-old - 8C_2w.15-day-old	-0.16	0.066	360	-2.407	**0.714**
	6C_1w.12-day-old - 6C_2w.12-day-old	0.11	0.066	360	1.654	**1**
	6C_1w.12-day-old - 4C_1w.12-day-old	0.13	0.066	360	1.955	**1**
	6C_1w.12-day-old - 4C_2w.12-day-old	0.34	0.066	360	5.114	**P < 0.001**

	6C_1w.12-day-old - 6C_1w.15-day-old	-0.28	0.066	360	-4.211	**0.002**
	6C_2w.12-day-old - 4C_1w.12-day-old	0.02	0.066	360	0.301	**1**
	6C_2w.12-day-old - 4C_2w.12-day-old	0.23	0.066	360	3.459	**0.03**
	6C_2w.12-day-old - 6C_2w.15-day-old	-0.32	0.066	360	-4.813	**P < 0.001**
	4C_1w.12-day-old - 4C_2w.12-day-old	0.21	0.066	360	3.159	**0.083**
	4C_1w.12-day-old - 4C_1w.15-day-old	0.03	0.066	360	0.451	**1**
	4C_2w.12-day-old - 4C_2w.15-day-old	-0.08	0.066	360	-1.203	**1**
	Control.15-day-old - 12C_1w.15-day-old	-0.03	0.066	360	-0.451	**1**
	Control.15-day-old - 10C_1w.15-day-old	-0.01	0.066	360	-0.15	**1**
	Control.15-day-old - 8C_1w.15-day-old	-0.01	0.066	360	-0.15	**1**
	Control.15-day-old - 8C_2w.15-day-old	0.1	0.066	360	1.504	**1**
	Control.15-day-old - 6C_1w.15-day-old	0.01	0.066	360	0.15	**1**
	Control.15-day-old - 6C_2w.15-day-old	0.08	0.066	360	1.203	**1**
	Control.15-day-old - 4C_1w.15-day-old	0.45	0.066	360	6.768	**P < 0.001**
	Control.15-day-old - 4C_2w.15-day-old	0.55	0.066	360	8.272	**P < 0.001**
	12C_1w.15-day-old - 10C_1w.15-day-old	0.02	0.066	360	0.301	**1**
	12C_1w.15-day-old - 8C_1w.15-day-old	0.02	0.066	360	0.301	**1**
	12C_1w.15-day-old - 8C_2w.15-day-old	0.13	0.066	360	1.955	**1**
	12C_1w.15-day-old - 6C_1w.15-day-old	0.04	0.066	360	0.602	**1**
	12C_1w.15-day-old - 6C_2w.15-day-old	0.11	0.066	360	1.654	**1**
	12C_1w.15-day-old - 4C_1w.15-day-old	0.48	0.066	360	7.22	**P < 0.001**
	12C_1w.15-day-old - 4C_2w.15-day-old	0.58	0.066	360	8.724	**P < 0.001**
	10C_1w.15-day-old - 8C_1w.15-day-old	0	0.066	360	0	**1**
	10C_1w.15-day-old - 8C_2w.15-day-old	0.11	0.066	360	1.654	**1**
	10C_1w.15-day-old - 6C_1w.15-day-old	0.02	0.066	360	0.301	**1**
	10C_1w.15-day-old - 6C_2w.15-day-old	0.09	0.066	360	1.354	**1**
	10C_1w.15-day-old - 4C_1w.15-day-old	0.46	0.066	360	6.919	**P < 0.001**
	10C_1w.15-day-old - 4C_2w.15-day-old	0.56	0.066	360	8.423	**P < 0.001**
	8C_1w.15-day-old - 8C_2w.15-day-old	0.11	0.066	360	1.654	**1**
	8C_1w.15-day-old - 6C_1w.15-day-old	0.02	0.066	360	0.301	**1**
	8C_1w.15-day-old - 6C_2w.15-day-old	0.09	0.066	360	1.354	**1**

**Species**	**Contrast**	**Estimate**	**SE**	**df**	**t.ratio**	**Significance**
	8C_1w.15-day-old - 4C_1w.15-day-old	0.46	0.066	360	6.919	**P < 0.001**
	8C_1w.15-day-old - 4C_2w.15-day-old	0.56	0.066	360	8.423	**P < 0.001**
	8C_2w.15-day-old - 6C_1w.15-day-old	-0.09	0.066	360	-1.354	**1**
	8C_2w.15-day-old - 6C_2w.15-day-old	-0.02	0.066	360	-0.301	**1**
	8C_2w.15-day-old - 4C_1w.15-day-old	0.35	0.066	360	5.264	**P < 0.001**

	8C_2w.15-day-old - 4C_2w.15-day-old	0.45	0.066	360	6.768	**P < 0.001**
	6C_1w.15-day-old - 6C_2w.15-day-old	0.07	0.066	360	1.053	**1**
	6C_1w.15-day-old - 4C_1w.15-day-old	0.44	0.066	360	6.618	**P < 0.001**
	6C_1w.15-day-old - 4C_2w.15-day-old	0.54	0.066	360	8.122	**P < 0.001**
	6C_2w.15-day-old - 4C_1w.15-day-old	0.37	0.066	360	5.565	**P < 0.001**
	6C_2w.15-day-old - 4C_2w.15-day-old	0.47	0.066	360	7.069	**P < 0.001**
	4C_1w.15-day-old - 4C_2w.15-day-old	0.1	0.066	360	1.504	**1**

Table 3. Results of the post hoc test with Holm correction for multiple comparisons on the linear regression with parasitoid emergence time as the response. The post hoc was performed through the R package lsmeans. Significant differences are given in bold.

Species	Contrast	Estimate	SE	df	t.ratio	Significance
Encarsia sophia	Control.10-day-old - 12C_1w.10-day-old	1.182	0.175	1273	6.751	**P < 0.001**
	Control.10-day-old - 12C_2w.10-day-old	2.344	0.176	1273	13.341	**P < 0.001**
	Control.10-day-old - 10C_1w.10-day-old	0.978	0.179	1273	5.469	**P < 0.001**
	Control.10-day-old - 10C_2w.10-day-old	1.991	0.179	1273	11.139	**P < 0.001**
	Control.10-day-old - 8C_1w.10-day-old	0.98	0.181	1273	5.425	**P < 0.001**
	Control.10-day-old - 8C_2w.10-day-old	1.332	0.193	1273	6.893	**P < 0.001**
	Control.10-day-old - 6C_1w.10-day-old	0.99	0.184	1273	5.373	**P < 0.001**
	Control.10-day-old - 6C_2w.10-day-old	0.203	0.202	1273	1.004	1
	Control.10-day-old - 4C_1w.10-day-old	0.941	0.194	1273	4.842	**P < 0.001**
	Control.10-day-old - 4C_2w.10-day-old	-1.256	0.293	1273	-4.283	**0.001**
	Control.10-day-old - Control.12-day-old	1.198	0.172	1273	6.966	**P < 0.001**
	12C_1w.10-day-old - 12C_2w.10-day-old	1.162	0.178	1273	6.515	**P < 0.001**
	12C_1w.10-day-old - 10C_1w.10-day-old	-0.205	0.181	1273	-1.131	1
	12C_1w.10-day-old - 10C_2w.10-day-old	0.808	0.181	1273	4.459	**P < 0.001**
	12C_1w.10-day-old - 8C_1w.10-day-old	-0.202	0.183	1273	-1.103	1
	12C_1w.10-day-old - 8C_2w.10-day-old	0.149	0.196	1273	0.764	1
	12C_1w.10-day-old - 6C_1w.10-day-old	-0.192	0.187	1273	-1.028	1
	12C_1w.10-day-old - 6C_2w.10-day-old	-0.979	0.205	1273	-4.782	**P < 0.001**
	12C_1w.10-day-old - 4C_1w.10-day-old	-0.242	0.197	1273	-1.23	1
	12C_1w.10-day-old - 4C_2w.10-day-old	-2.438	0.295	1273	-8.272	**P < 0.001**
	12C_1w.10-day-old - 12C_1w.12-day-old	1.978	0.176	1273	11.23	**P < 0.001**
	12C_2w.10-day-old - 10C_1w.10-day-old	-1.367	0.182	1273	-7.516	**P < 0.001**
	12C_2w.10-day-old - 10C_2w.10-day-old	-0.353	0.182	1273	-1.943	1
	12C_2w.10-day-old - 8C_1w.10-day-old	-1.364	0.184	1273	-7.422	**P < 0.001**
	12C_2w.10-day-old - 8C_2w.10-day-old	-1.012	0.196	1273	-5.163	**P < 0.001**
	12C_2w.10-day-old - 6C_1w.10-day-old	-1.354	0.187	1273	-7.226	**P < 0.001**
	12C_2w.10-day-old - 6C_2w.10-day-old	-2.141	0.205	1273	-10.432	**P < 0.001**
	12C_2w.10-day-old - 4C_1w.10-day-old	-1.404	0.197	1273	-7.121	**P < 0.001**
	12C_2w.10-day-old - 4C_2w.10-day-old	-3.6	0.295	1273	-12.199	**P < 0.001**
	10C_1w.10-day-old - 10C_2w.10-day-old	1.013	0.185	1273	5.485	**P < 0.001**
	10C_1w.10-day-old - 8C_1w.10-day-old	0.003	0.187	1273	0.015	1
**Species**	**Contrast**	**Estimate**	**SE**	**df**	**t.ratio**	**Significance**
	10C_1w.10-day-old - 8C_2w.10-day-old	0.354	0.199	1273	1.783	1

	10C_1w.10-day-old - 6C_1w.10-day-old	0.013	0.19	1273	0.068	1
	10C_1w.10-day-old - 6C_2w.10-day-old	-0.774	0.208	1273	-3.726	**0.008**
	10C_1w.10-day-old - 4C_1w.10-day-old	-0.037	0.2	1273	-0.185	1
	10C_1w.10-day-old - 4C_2w.10-day-old	-2.233	0.297	1273	-7.522	**P < 0.001**
	10C_1w.10-day-old - 10C_1w.12-day-old	2.02	0.179	1273	11.269	**P < 0.001**
	8C_1w.10-day-old - 8C_2w.10-day-old	0.352	0.201	1273	1.753	1
	8C_1w.10-day-old - 6C_1w.10-day-old	0.01	0.192	1273	0.053	1
	8C_1w.10-day-old - 6C_2w.10-day-old	-0.777	0.21	1273	-3.708	**0.008**
	8C_1w.10-day-old - 4C_1w.10-day-old	-0.04	0.202	1273	-0.197	1
	8C_1w.10-day-old - 4C_2w.10-day-old	-2.236	0.298	1273	-7.501	**P < 0.001**
	8C_1w.10-day-old - 8C_1w.12-day-old	1.241	0.18	1273	6.904	**P < 0.001**
	8C_2w.10-day-old - 6C_1w.10-day-old	-0.341	0.204	1273	-1.675	1
	8C_2w.10-day-old - 6C_2w.10-day-old	-1.129	0.22	1273	-5.121	**P < 0.001**
	8C_2w.10-day-old - 4C_1w.10-day-old	-0.391	0.213	1273	-1.838	1
	8C_2w.10-day-old - 4C_2w.10-day-old	-2.588	0.306	1273	-8.461	**P < 0.001**
	8C_2w.10-day-old - 8C_2w.12-day-old	0.082	0.216	1273	0.38	1
	6C_1w.10-day-old - 4C_1w.10-day-old	-0.05	0.205	1273	-0.243	1
	6C_1w.10-day-old - 4C_2w.10-day-old	-2.246	0.3	1273	-7.479	**P < 0.001**
	6C_1w.10-day-old - 6C_1w.12-day-old	0.873	0.185	1273	4.71	**P < 0.001**
	6C_2w.10-day-old - 4C_1w.10-day-old	0.737	0.221	1273	3.332	**0.03**
	6C_2w.10-day-old - 4C_2w.10-day-old	-1.459	0.312	1273	-4.68	**P < 0.001**
	6C_2w.10-day-old - 6C_2w.12-day-old	0.878	0.236	1273	3.714	**0.008**
	4C_1w.10-day-old - 4C_2w.10-day-old	-2.196	0.307	1273	-7.166	**P < 0.001**
	4C_1w.10-day-old - 4C_1w.12-day-old	0.278	0.236	1273	1.178	1
	4C_2w.10-day-old - 4C_2w.12-day-old	1.786	0.403	1273	4.43	**P < 0.001**
	Control.12-day-old - 12C_1w.12-day-old	1.963	0.173	1273	11.342	**P < 0.001**
	Control.12-day-old - 10C_1w.12-day-old	1.799	0.173	1273	10.427	**P < 0.001**
	Control.12-day-old - 8C_1w.12-day-old	1.023	0.171	1273	5.983	**P < 0.001**
	Control.12-day-old - 8C_2w.12-day-old	0.216	0.197	1273	1.095	**1**
	Control.12-day-old - 6C_1w.12-day-old	0.665	0.173	1273	3.843	**0.005**
	Control.12-day-old - 6C_2w.12-day-old	-0.117	0.211	1273	-0.554	**1**
	Control.12-day-old - 4C_1w.12-day-old	0.02	0.218	1273	0.093	**1**
	Control.12-day-old - 4C_2w.12-day-old	-0.668	0.326	1273	-2.052	**1**
	12C1w.12-day-old - 10C_1w.12-day-old	-0.164	0.174	1273	-0.941	**1**
**Species**	**Contrast**	**Estimate**	**SE**	**df**	**t.ratio**	**Significance**
	12C_1w.12-day-old - 8C_1w.12-day-old	-0.939	0.173	1273	-5.444	**P < 0.001**
	12C_1w.12-day-old - 8C_2w.12-day-old	-1.747	0.198	1273	-8.803	**P < 0.001**

	12C_1w.12-day-old - 6C_1w.12-day-old	-1.298	0.175	1273	-7.434	**P < 0.001**
	12C_1w.12-day-old - 6C_2w.12-day-old	-2.079	0.212	1273	-9.803	**P < 0.001**
	12C_1w.12-day-old - 4C_1w.12-day-old	-1.942	0.219	1273	-8.861	**P < 0.001**
	12C_1w.12-day-old - 4C_2w.12-day-old	-2.631	0.327	1273	-8.057	**P < 0.001**
	10C_1w.12-day-old - 8C_1w.12-day-old	-0.776	0.172	1273	-4.509	**P < 0.001**
	10C_1w.12-day-old - 8C_2w.12-day-old	-1.583	0.198	1273	-7.996	**P < 0.001**
	10C_1w.12-day-old - 6C_1w.12-day-old	-1.134	0.174	1273	-6.515	**P < 0.001**
	10C_1w.12-day-old - 6C_2w.12-day-old	-1.916	0.212	1273	-9.049	**P < 0.001**
	10C_1w.12-day-old - 4C_1w.12-day-old	-1.779	0.219	1273	-8.129	**P < 0.001**
	10C_1w.12-day-old - 4C_2w.12-day-old	-2.467	0.326	1273	-7.561	**P < 0.001**
	8C_1w.12-day-old - 8C_2w.12-day-old	-0.807	0.197	1273	-4.105	**0.002**
	8C_1w.12-day-old - 6C_1w.12-day-old	-0.358	0.173	1273	-2.076	**1**
	8C_1w.12-day-old - 6C_2w.12-day-old	-1.14	0.21	1273	-5.416	**P < 0.001**
	8C_1w.12-day-old - 4C_1w.12-day-old	-1.003	0.218	1273	-4.609	**P < 0.001**
	8C_1w.12-day-old - 4C_2w.12-day-old	-1.692	0.326	1273	-5.197	**P < 0.001**
	8C_2w.12-day-old - 6C_1w.12-day-old	0.449	0.198	1273	2.264	**0.736**
	8C_2w.12-day-old - 6C_2w.12-day-old	-0.333	0.232	1273	-1.433	**1**
	8C_2w.12-day-old - 4C_1w.12-day-old	-0.195	0.239	1273	-0.819	**1**
	8C_2w.12-day-old - 4C_2w.12-day-old	-0.884	0.34	1273	-2.601	**0.301**
	6C_1w.12-day-old - 6C_2w.12-day-old	-0.782	0.212	1273	-3.686	**0.008**
	6C_1w.12-day-old - 4C_1w.12-day-old	-0.645	0.219	1273	-2.941	**0.11**
	6C_1w.12-day-old - 4C_2w.12-day-old	-1.333	0.327	1273	-4.083	**0.002**
	6C_2w.12-day-old - 4C_1w.12-day-old	0.137	0.25	1273	0.548	**1**
	6C_2w.12-day-old - 4C_2w.12-day-old	-0.551	0.348	1273	-1.584	**1**
	4C_1w.12-day-old - 4C_2w.12-day-old	-0.689	0.352	1273	-1.954	**1**
Eretmocerus hayati	Control.12-day-old - 12C_1w.12-day-old	0.673	0.108	1261	6.234	**P < 0.001**
	Control.12-day-old - 10C_1w.12-day-old	0.607	0.109	1261	5.585	**P < 0.001**
	Control.12-day-old - 10C_2w.12-day-old	2.408	0.112	1261	21.526	**P < 0.001**
	Control.12-day-old - 8C_1w.12-day-old	0.329	0.119	1261	2.77	**0.165**
	Control.12-day-old - 8C_2w.12-day-old	1.362	0.121	1261	11.288	**P < 0.001**
	Control.12-day-old - 6C_1w.12-day-old	0.438	0.123	1261	3.569	**0.014**
	Control.12-day-old - 6C_2w.12-day-old	1.093	0.132	1261	8.307	**P < 0.001**
	Control.12-day-old - 4C_1w.12-day-old	0.432	0.134	1261	3.237	**0.042**
**Species**	**Contrast**	**Estimate**	**SE**	**df**	**t.ratio**	**Significance**
	Control.12-day-old - 4C_2w.12-day-old	0.47	0.171	1261	2.743	**0.173**
	Control.12-day-old - Control.15-day-old	1.226	0.109	1261	11.251	**P < 0.001**
	12C_1w.12-day-old - 10C_1w.12-day-old	-0.066	0.109	1261	-0.609	**1**

	12C_1w.12-day-old - 10C_2w.12-day-old	1.734	0.112	1261	15.426	**P < 0.001**
	12C_1w.12-day-old - 8C_1w.12-day-old	-0.344	0.119	1261	-2.879	**0.122**
	12C_1w.12-day-old - 8C_2w.12-day-old	0.689	0.121	1261	5.684	**P < 0.001**
	12C_1w.12-day-old - 6C_1w.12-day-old	-0.236	0.123	1261	-1.913	**1**
	12C_1w.12-day-old - 6C_2w.12-day-old	0.42	0.132	1261	3.176	**0.05**
	12C_1w.12-day-old - 4C_1w.12-day-old	-0.241	0.134	1261	-1.797	**1**
	12C_1w.12-day-old - 4C_2w.12-day-old	-0.203	0.172	1261	-1.182	**1**
	12C_1w.12-day-old - 12C_1w.15-day-old	1.695	0.109	1261	15.604	**P < 0.001**
	10C_1w.12-day-old - 10C_2w.12-day-old	1.801	0.113	1261	15.93	**P < 0.001**
	10C_1w.12-day-old - 8C_1w.12-day-old	-0.277	0.12	1261	-2.311	**0.503**
	10C_1w.12-day-old - 8C_2w.12-day-old	0.756	0.122	1261	6.203	**P < 0.001**
	10C_1w.12-day-old - 6C_1w.12-day-old	-0.169	0.124	1261	-1.367	**1**
	10C_1w.12-day-old - 6C_2w.12-day-old	0.486	0.133	1261	3.666	**0.01**
	10C_1w.12-day-old - 4C_1w.12-day-old	-0.174	0.135	1261	-1.296	**1**
	10C_1w.12-day-old - 4C_2w.12-day-old	-0.137	0.172	1261	-0.793	**1**
	10C_1w.12-day-old - 10C_1w.15-day-old	1.568	0.109	1261	14.353	**P < 0.001**
	10C_2w.12-day-old - 8C_1w.12-day-old	-2.078	0.123	1261	-16.903	**P < 0.001**
	10C_2w.12-day-old - 8C_2w.12-day-old	-1.045	0.125	1261	-8.384	**P < 0.001**
	10C_2w.12-day-old - 6C_1w.12-day-old	-1.97	0.127	1261	-15.568	**P < 0.001**
	10C_2w.12-day-old - 6C_2w.12-day-old	-1.315	0.135	1261	-9.724	**P < 0.001**
	10C_2w.12-day-old - 4C_1w.12-day-old	-1.975	0.137	1261	-14.4	**P < 0.001**
	10C_2w.12-day-old - 4C_2w.12-day-old	-1.938	0.174	1261	-11.122	**P < 0.001**
	8C_1w.12-day-old - 8C_2w.12-day-old	1.033	0.131	1261	7.883	**P < 0.001**
	8C_1w.12-day-old - 6C_1w.12-day-old	0.108	0.133	1261	0.815	**1**
	8C_1w.12-day-old - 6C_2w.12-day-old	0.763	0.141	1261	5.41	**P < 0.001**
	8C_1w.12-day-old - 4C_1w.12-day-old	0.103	0.143	1261	0.721	**1**
	8C_1w.12-day-old - 4C_2w.12-day-old	0.141	0.179	1261	0.787	**1**
	8C_1w.12-day-old - 8C_1w.15-day-old	1.762	0.12	1261	14.673	**P < 0.001**
	8C_2w.12-day-old - 6C_1w.12-day-old	-0.925	0.134	1261	-6.879	**P < 0.001**
	8C_2w.12-day-old - 6C_2w.12-day-old	-0.27	0.143	1261	-1.891	**1**
	8C_2w.12-day-old - 4C_1w.12-day-old	-0.93	0.144	1261	-6.437	**P < 0.001**
	8C_2w.12-day-old - 4C_2w.12-day-old	-0.892	0.18	1261	-4.956	**P < 0.001**
**Species**	**Contrast**	**Estimate**	**SE**	**df**	**t.ratio**	**Significance**
	8C_2w.12-day-old - 8C_2w.15-day-old	0.431	0.125	1261	3.436	**0.023**
	6C_1w.12-day-old - 6C_2w.12-day-old	0.655	0.144	1261	4.541	**P < 0.001**
	6C_1w.12-day-old - 4C_1w.12-day-old	-0.005	0.146	1261	-0.036	**1**
	6C_1w.12-day-old - 4C_2w.12-day-old	0.033	0.181	1261	0.179	**1**

	6C_1w.12-day-old - 6C_1w.15-day-old	1.702	0.124	1261	13.695	**P < 0.001**
	6C_2w.12-day-old - 4C_1w.12-day-old	-0.66	0.154	1261	-4.298	**0.001**
	6C_2w.12-day-old - 4C_2w.12-day-old	-0.623	0.188	1261	-3.32	**0.032**
	6C_2w.12-day-old - 6C_2w.15-day-old	0.354	0.135	1261	2.617	**0.242**
	4C_1w.12-day-old - 4C_2w.12-day-old	0.038	0.189	1261	0.2	**1**
	4C_1w.12-day-old - 4C_1w.15-day-old	2.253	0.158	1261	14.231	**P < 0.001**
	4C_2w.12-day-old - 4C_2w.15-day-old	0.707	0.202	1261	3.496	**0.019**
	Control.15-day-old - 12C_1w.15-day-old	1.142	0.11	1261	10.424	**P < 0.001**
	Control.15-day-old - 10C_1w.15-day-old	0.949	0.11	1261	8.661	**P < 0.001**
	Control.15-day-old - 8C_1w.15-day-old	0.865	0.11	1261	7.848	**P < 0.001**
	Control.15-day-old - 8C_2w.15-day-old	0.567	0.114	1261	4.969	**P < 0.001**
	Control.15-day-old - 6C_1w.15-day-old	0.914	0.111	1261	8.245	**P < 0.001**
	Control.15-day-old - 6C_2w.15-day-old	0.221	0.113	1261	1.947	**1**
	Control.15-day-old - 4C_1w.15-day-old	1.459	0.138	1261	10.558	**P < 0.001**
	Control.15-day-old - 4C_2w.15-day-old	-0.049	0.153	1261	-0.319	**1**
	12C_1w.15-day-old - 10C_1w.15-day-old	-0.193	0.109	1261	-1.778	**1**
	12C_1w.15-day-old - 8C_1w.15-day-old	-0.277	0.109	1261	-2.538	**0.282**
	12C_1w.15-day-old - 8C_2w.15-day-old	-0.575	0.113	1261	-5.078	**P < 0.001**
	12C_1w.15-day-old - 6C_1w.15-day-old	-0.228	0.11	1261	-2.077	**0.836**
	12C_1w.15-day-old - 6C_2w.15-day-old	-0.922	0.112	1261	-8.196	**P < 0.001**
	12C_1w.15-day-old - 4C_1w.15-day-old	0.317	0.137	1261	2.304	**0.503**
	12C_1w.15-day-old - 4C_2w.15-day-old	-1.191	0.152	1261	-7.817	**P < 0.001**
	10C_1w.15-day-old - 8C_1w.15-day-old	-0.084	0.109	1261	-0.77	**1**
	10C_1w.15-day-old - 8C_2w.15-day-old	-0.382	0.113	1261	-3.372	**0.028**
	10C_1w.15-day-old - 6C_1w.15-day-old	-0.035	0.11	1261	-0.319	**1**
	10C_1w.15-day-old - 6C_2w.15-day-old	-0.728	0.112	1261	-6.478	**P < 0.001**
	10C_1w.15-day-old - 4C_1w.15-day-old	0.51	0.137	1261	3.71	**0.009**
	10C_1w.15-day-old - 4C_2w.15-day-old	-0.998	0.152	1261	-6.549	**P < 0.001**
	8C_1w.15-day-old - 8C_2w.15-day-old	-0.298	0.114	1261	-2.615	**0.242**
	8C_1w.15-day-old - 6C_1w.15-day-old	0.049	0.111	1261	0.443	**1**
	8C_1w.15-day-old - 6C_2w.15-day-old	-0.644	0.113	1261	-5.699	**P < 0.001**

**Species**	**Contrast**	**Estimate**	**SE**	**df**	**t.ratio**	**Significance**
	8C_1w.15-day-old - 4C_1w.15-day-old	0.594	0.138	1261	4.306	**0.001**
	8C_1w.15-day-old - 4C_2w.15-day-old	-0.914	0.153	1261	-5.98	**P < 0.001**
	8C_2w.15-day-old - 6C_1w.15-day-old	0.347	0.114	1261	3.029	**0.08**
	8C_2w.15-day-old - 6C_2w.15-day-old	-0.347	0.117	1261	-2.964	**0.096**
	8C_2w.15-day-old - 4C_1w.15-day-old	0.892	0.141	1261	6.32	**P < 0.001**

	8C_2w.15-day-old - 4C_2w.15-day-old	-0.616	0.156	1261	-3.957	**0.003**
	6C_1w.15-day-old - 6C_2w.15-day-old	-0.693	0.114	1261	-6.098	**P < 0.001**
	6C_1w.15-day-old - 4C_1w.15-day-old	0.545	0.138	1261	3.936	**0.004**
	6C_1w.15-day-old - 4C_2w.15-day-old	-0.963	0.153	1261	-6.281	**P < 0.001**
	6C_2w.15-day-old - 4C_1w.15-day-old	1.238	0.14	1261	8.815	**P < 0.001**
	6C_2w.15-day-old - 4C_2w.15-day-old	-0.269	0.155	1261	-1.737	**1**
	4C_1w.15-day-old - 4C_2w.15-day-old	-1.508	0.174	1261	-8.66	**P < 0.001**

Table 4. Results of the post hoc test with Holm correction for multiple comparisons on the linear regression with adult parasitoid longevity as the response. The post hoc was performed through the R package lsmeans. Significant differences are given in bold.

Species	Contrast	Estimate	SE	df	t.ratio	Significance
Encarsia sophia	Control - 12C_1w	-0.446	1.194	528	-0.374	1
	Control - 12C_2w	-2.935	1.558	528	-1.884	1
	Control - 10C_1w	-0.35	1.189	528	-0.294	1
	Control - 10C_2w	-1.265	1.579	528	-0.801	1
	Control - 8C_1w	1.317	1.189	528	1.107	1
	Control - 8C_2w	1.182	1.235	528	0.957	1
	Control - 6C_1w	1.15	1.189	528	0.967	1
	Control - 6C_2w	0.38	1.247	528	0.305	1
	Control - 4C_1w	2.846	1.194	528	2.383	0.666
	Control - 4C_2w	7.725	1.476	528	5.233	**P < 0.001**
	12C_1w - 12C_2w	-2.488	1.563	528	-1.592	1
	12C_1w - 10C_1w	0.096	1.194	528	0.081	1
	12C_1w - 10C_2w	-0.819	1.584	528	-0.517	1
	12C_1w - 8C_1w	1.763	1.194	528	1.476	1
	12C_1w - 8C_2w	1.629	1.24	528	1.313	1
	12C_1w - 6C_1w	1.596	1.194	528	1.336	1
	12C_1w - 6C_2w	0.826	1.252	528	0.66	1
	12C_1w - 4C_1w	3.293	1.2	528	2.745	0.275
	12C_1w - 4C_2w	8.171	1.481	528	5.519	**P < 0.001**
	12C_2w - 10C_1w	2.585	1.558	528	1.659	1
	12C_2w - 10C_2w	1.669	1.825	528	0.915	1
	12C_2w - 8C_1w	4.251	1.558	528	2.729	0.283
	12C_2w - 8C_2w	4.117	1.585	528	2.598	0.384
	12C_2w - 6C_1w	4.085	1.558	528	2.622	0.369
	12C_2w - 6C_2w	3.315	1.603	528	2.068	1
	12C_2w - 4C_1w	5.781	1.561	528	3.704	**0.011**
	12C_2w - 4C_2w	10.659	1.771	528	6.018	**P < 0.001**
	10C_1w - 10C_2w	-0.915	1.579	528	-0.58	1
	10C_1w - 8C_1w	1.667	1.189	528	1.401	1
	10C_1w - 8C_2w	1.532	1.235	528	1.241	1
	10C_1w - 6C_1w	1.5	1.189	528	1.261	1
	10C_1w - 6C_2w	0.73	1.247	528	0.585	1

	10C_1w - 4C_1w	3.196	1.194	528	2.676	0.323
	10C_1w - 4C_2w	8.075	1.476	528	5.47	**P < 0.001**
	10C_2w - 8C_1w	2.582	1.579	528	1.635	1
	10C_2w - 8C_2w	2.448	1.605	528	1.525	1
	10C_2w - 6C_1w	2.415	1.579	528	1.53	1
	10C_2w - 6C_2w	1.645	1.623	528	1.014	1
	10C_2w - 4C_1w	4.112	1.582	528	2.6	0.384
	10C_2w - 4C_2w	8.99	1.79	528	5.023	**P < 0.001**
	8C_1w - 8C_2w	-0.134	1.235	528	-0.109	1
	8C_1w - 6C_1w	-0.167	1.189	528	-0.14	1
	8C_1w - 6C_2w	-0.937	1.247	528	-0.751	1
	8C_1w - 4C_1w	1.53	1.194	528	1.281	1
	8C_1w - 4C_2w	6.408	1.476	528	4.341	**0.001**
	8C_2w - 6C_1w	-0.032	1.235	528	-0.026	1
	8C_2w - 6C_2w	-0.802	1.291	528	-0.621	1
	8C_2w - 4C_1w	1.664	1.24	528	1.342	1
	8C_2w - 4C_2w	6.542	1.511	528	4.331	**0.001**
	6C_1w - 6C_2w	-0.77	1.247	528	-0.617	1
	6C_1w - 4C_1w	1.696	1.194	528	1.42	1
	6C_1w - 4C_2w	6.575	1.476	528	4.454	0.001
	6C_2w - 4C_1w	2.466	1.252	528	1.969	1
	6C_2w - 4C_2w	7.345	1.523	528	4.821	P < 0.001
	4C_1w - 4C_2w	4.878	1.48	528	3.296	0.047
Eretmocerus hayati	Control - 12C_1w	0.235	0.402	943	0.585	1
	Control - 10C_1w	0.502	0.402	943	1.247	1
	Control - 10C_2w	2.786	0.514	943	5.422	P < 0.001
	Control - 8C_1w	0.627	0.415	943	1.508	1
	Control - 8C_2w	3.994	0.416	943	9.592	P < 0.001
	Control - 6C_1w	0.655	0.409	943	1.599	1
	Control - 6C_2w	4.029	0.429	943	9.396	P < 0.001
	Control - 4C_1w	3.74	0.462	943	8.091	P < 0.001
	Control - 4C_2w	5.244	0.524	943	10.01	P < 0.001
	12C_1w - 10C_1w	0.267	0.401	943	0.666	1
	12C_1w - 10C_2w	2.551	0.513	943	4.974	P < 0.001
**Species**	**Contrast**	**Estimate**	**SE**	**df**	**t.ratio**	**Significance**
	12C_1w - 8C_1w	0.392	0.414	943	0.946	1
	12C_1w - 8C_2w	3.759	0.415	943	9.064	P < 0.001

	12C_1w - 6C_1w	0.42	0.408	943	1.029	1
	12C_1w - 6C_2w	3.793	0.427	943	8.881	P < 0.001
	12C_1w - 4C_1w	3.505	0.461	943	7.606	P < 0.001
	12C_1w - 4C_2w	5.009	0.523	943	9.586	P < 0.001
	10C_1w - 10C_2w	2.284	0.513	943	4.454	P < 0.001
	10C_1w - 8C_1w	0.125	0.414	943	0.302	1
	10C_1w - 8C_2w	3.492	0.415	943	8.421	P < 0.001
	10C_1w - 6C_1w	0.153	0.408	943	0.375	1
	10C_1w - 6C_2w	3.527	0.427	943	8.257	P < 0.001
	10C_1w - 4C_1w	3.239	0.461	943	7.027	P < 0.001
	10C_1w - 4C_2w	4.742	0.523	943	9.076	P < 0.001
	10C_2w - 8C_1w	-2.159	0.524	943	-4.118	0.001
	10C_2w - 8C_2w	1.208	0.525	943	2.301	0.362
	10C_2w - 6C_1w	-2.131	0.52	943	-4.099	0.001
	10C_2w - 6C_2w	1.242	0.539	943	2.307	0.362
	10C_2w - 4C_1w	0.954	0.56	943	1.703	1
	10C_2w - 4C_2w	2.458	0.615	943	3.997	0.001
	8C_1w - 8C_2w	3.368	0.428	943	7.875	P < 0.001
	8C_1w - 6C_1w	0.028	0.421	943	0.067	1
	8C_1w - 6C_2w	3.402	0.439	943	7.752	P < 0.001
	8C_1w - 4C_1w	3.114	0.472	943	6.596	P < 0.001
	8C_1w - 4C_2w	4.618	0.533	943	8.666	P < 0.001
	8C_2w - 6C_1w	-3.34	0.422	943	-7.922	P < 0.001
	8C_2w - 6C_2w	0.034	0.44	943	0.078	1
	8C_2w - 4C_1w	-0.254	0.473	943	-0.536	1
	8C_2w - 4C_2w	1.25	0.533	943	2.344	0.347
	6C_1w - 6C_2w	3.374	0.433	943	7.783	P < 0.001
	6C_1w - 4C_1w	3.086	0.467	943	6.604	P < 0.001
	6C_1w - 4C_2w	4.59	0.528	943	8.695	P < 0.001
	6C_2w - 4C_1w	-0.288	0.484	943	-0.596	1
	6C_2w - 4C_2w	1.216	0.543	943	2.24	0.38
	4C_1w - 4C_2w	1.504	0.57	943	2.637	0.162

Table 5. Results of the post hoc test with Holm correction for multiple comparisons on the linear regression with adult body size as the response. The post hoc was performed through the R package lsmeans. Significant differences are given in bold.

Species	Contrast	Estimate	SE	df	t.ratio	Significance
Encarsia sophia	Control - 12C_1w	0.137	0.195	362	0.704	1
	Control - 10C_1w	0.1	0.195	362	0.512	1
	Control - 10C_2w	0.25	0.239	362	1.045	1
	Control - 8C_1w	0.062	0.195	362	0.32	1
	Control - 8C_2w	0.312	0.195	362	1.6	1
	Control - 6C_1w	0.262	0.195	362	1.344	1
	Control - 6C_2w	0.387	0.195	362	1.984	1
	Control - 4C_1w	0.35	0.195	362	1.792	1
	Control - 4C_2w	0.672	0.207	362	3.244	0.058
	12C_1w - 10C_1w	-0.037	0.195	362	-0.192	1
	12C_1w - 10C_2w	0.113	0.239	362	0.47	1
	12C_1w - 8C_1w	-0.075	0.195	362	-0.384	1
	12C_1w - 8C_2w	0.175	0.195	362	0.896	1
	12C_1w - 6C_1w	0.125	0.195	362	0.64	1
	12C_1w - 6C_2w	0.25	0.195	362	1.28	1
	12C_1w - 4C_1w	0.213	0.195	362	1.088	1
	12C_1w - 4C_2w	0.534	0.207	362	2.58	0.432
	10C_1w - 10C_2w	0.15	0.239	362	0.627	1
	10C_1w - 8C_1w	-0.037	0.195	362	-0.192	1
	10C_1w - 8C_2w	0.212	0.195	362	1.088	1
	10C_1w - 6C_1w	0.163	0.195	362	0.832	1
	10C_1w - 6C_2w	0.288	0.195	362	1.472	1
	10C_1w - 4C_1w	0.25	0.195	362	1.28	1
	10C_1w - 4C_2w	0.572	0.207	362	2.761	0.26
	10C_2w - 8C_1w	-0.188	0.239	362	-0.784	1
	10C_2w - 8C_2w	0.062	0.239	362	0.261	1
	10C_2w - 6C_1w	0.012	0.239	362	0.052	1
	10C_2w - 6C_2w	0.137	0.239	362	0.575	1
	10C_2w - 4C_1w	0.1	0.239	362	0.418	1
	10C_2w - 4C_2w	0.422	0.249	362	1.695	1
	8C_1w - 8C_2w	0.25	0.195	362	1.28	1
**Species**	**Contrast**	**Estimate**	**SE**	**df**	**t.ratio**	**Significance**
	8C_1w - 6C_1w	0.2	0.195	362	1.024	1

	8C_1w - 6C_2w	0.325	0.195	362	1.664	1
	8C_1w - 4C_1w	0.288	0.195	362	1.472	1
	8C_1w - 4C_2w	0.609	0.207	362	2.942	0.153
	8C_2w - 6C_1w	-0.05	0.195	362	-0.256	1
	8C_2w - 6C_2w	0.075	0.195	362	0.384	1
	8C_2w - 4C_1w	0.038	0.195	362	0.192	1
	8C_2w - 4C_2w	0.359	0.207	362	1.735	1
	6C_1w - 6C_2w	0.125	0.195	362	0.64	1
	6C_1w - 4C_1w	0.087	0.195	362	0.448	1
	6C_1w - 4C_2w	0.409	0.207	362	1.976	1
	6C_2w - 4C_1w	-0.038	0.195	362	-0.192	1
	6C_2w - 4C_2w	0.284	0.207	362	1.373	1
	4C_1w - 4C_2w	0.322	0.207	362	1.554	1
Eretmocerus hayati	Control - 12C_1w	0.144	0.147	709	0.981	1
	Control - 10C_1w	0.287	0.147	709	1.961	1
	Control - 10C_2w	0.717	0.186	709	3.856	**0.005**
	Control - 8C_1w	0.287	0.147	709	1.961	1
	Control - 8C_2w	0.613	0.148	709	4.152	**0.002**
	Control - 6C_1w	0.387	0.147	709	2.644	0.243
	Control - 6C_2w	0.792	0.148	709	5.369	P < 0.001
	Control - 4C_1w	0.466	0.148	709	3.148	0.057
	Control - 4C_2w	0.965	0.167	709	5.772	P < 0.001
	12C_1w - 10C_1w	0.144	0.147	709	0.981	1
	12C_1w - 10C_2w	0.574	0.186	709	3.083	0.068
	12C_1w - 8C_1w	0.144	0.147	709	0.981	1
	12C_1w - 8C_2w	0.469	0.148	709	3.178	0.053
	12C_1w - 6C_1w	0.244	0.147	709	1.663	1
	12C_1w - 6C_2w	0.648	0.148	709	4.394	0.001
	12C_1w - 4C_1w	0.322	0.148	709	2.177	0.726
	12C_1w - 4C_2w	0.821	0.167	709	4.912	P < 0.001
	10C_1w - 10C_2w	0.43	0.186	709	2.311	0.592
	10C_1w - 8C_1w	0	0.147	709	0	1
	10C_1w - 8C_2w	0.325	0.148	709	2.203	0.726
	10C_1w - 6C_1w	0.1	0.147	709	0.682	1
**Species**	**Contrast**	**Estimate**	**SE**	**df**	**t.ratio**	**Significance**
	10C_1w - 6C_2w	0.504	0.148	709	3.42	0.024
	10C_1w - 4C_1w	0.178	0.148	709	1.206	1

	10C_1w - 4C_2w	0.678	0.167	709	4.053	0.002
	10C_2w - 8C_1w	-0.43	0.186	709	-2.311	0.592
	10C_2w - 8C_2w	-0.105	0.187	709	-0.561	1
	10C_2w - 6C_1w	-0.33	0.186	709	-1.773	1
	10C_2w - 6C_2w	0.075	0.187	709	0.399	1
	10C_2w - 4C_1w	-0.251	0.187	709	-1.344	1
	10C_2w - 4C_2w	0.248	0.203	709	1.217	1
	8C_1w - 8C_2w	0.325	0.148	709	2.203	0.726
	8C_1w - 6C_1w	0.1	0.147	709	0.682	1
	8C_1w - 6C_2w	0.504	0.148	709	3.42	0.024
	8C_1w - 4C_1w	0.178	0.148	709	1.206	1
	8C_1w - 4C_2w	0.678	0.167	709	4.053	0.002
	8C_2w - 6C_1w	-0.225	0.148	709	-1.525	1
	8C_2w - 6C_2w	0.179	0.148	709	1.209	1
	8C_2w - 4C_1w	-0.147	0.149	709	-0.984	1
	8C_2w - 4C_2w	0.353	0.168	709	2.099	0.796
	6C_1w - 6C_2w	0.404	0.148	709	2.742	0.188
	6C_1w - 4C_1w	0.078	0.148	709	0.53	1
	6C_1w - 4C_2w	0.578	0.167	709	3.454	0.022
	6C_2w - 4C_1w	-0.326	0.149	709	-2.189	0.726
	6C_2w - 4C_2w	0.173	0.168	709	1.03	1
	4C_1w - 4C_2w	0.499	0.168	709	2.962	0.098

Table 6. Effect on hind tibia length (μm) in *Encarsia sophia* after pupal exposure to cold storage at different temperatures and duration. Data are means ± SE.

Storage treatment	Tibia length of females emerging from	
	10-day-old pupae	12-day-old pupae
Control (26 °C)	153.61 ± 2.14aA	154.72 ± 1.77aA
12°C / 1 week	152.50 ± 2.69aA	152.78 ± 2.23aA
12 °C / 2 weeks	⊗ [1]	⊗
10°C / 1 week	153.61 ± 2.39aA	152.50 ± 1.78aA
10°C / 2 weeks	151.39 ± 2.01a	⊗
8°C / 1 week	153.89 ± 2.58aA	153.06 ± 1.95aA
8°C / 2 weeks	150.28 ± 2.03aA	151.11 ± 2.30aA
6°C / 1 week	151.39 ± 2.48aA	151.11 ± 2.23aA
6°C / 2 weeks	149.17 ± 2.29aA	150.56 ± 1.56aA
4°C / 1 weeks	150.56 ± 2.76aA	150.00 ± 1.93aA
4°C / 2 weeks	148.77 ± 2.09aA	144.05 ± 2.36aA

[1] The symbol (⊗) referred discarded data as the pupae were emerged during cold storage treatment.

Table S7 Effect on hind tibia length (µm) in *Eretmocerus hayati* after pupal exposure to cold storage at different temperatures and duration. Data are means ± SE.

Storage treatment	Tibia length of females emerging from		Tibia length of males emerging from	
	12-day-old pupae	15-day-old pupae	12-day-old pupae	15-day-old pupae
Control (26 °C)	188.1 ± 1.9Aa	190.3 ± 2.0Aa	193.9 ± 2.3Aa	195.3 ± 2.7Aa
12°C / 1 week	187.2 ± 2.2Aab	187.8 ± 1.8Aa	191.7 ± 2.4Aab	194.4 ± 2.5Aa
10°C / 1 week	186.1 ± 2.2Aabc	186.9 ± 1.7Aa	191.1 ± 2.8Aabc	190.6 ± 2.5Aa
10°C / 2 weeks	180.1 ± 2.8bcd	⊗ [1]	184.2 ± 2.5cd	⊗
8°C / 1 week	185.3 ± 1.7Aabc	186.6 ± 1.6Aa	190.6 ± 2.7Aabc	192.2 ± 2.0Aa
8°C / 2 weeks	179.5 ± 3.4Abcd	186.4 ± 2.0Aa	185.4 ± 3.4Acd	188.9 ± 1.3Aa
6°C / 1 week	182.8 ± 2.2Aabc	185.8 ± 1.8Aa	189.2 ± 1.9Aabc	192.5 ± 3.4Aa
6°C / 2 weeks	176.3 ± 2.6Bcd	186.7 ± 2.0Aa	182.5 ± 2.3Acd	186.7 ± 1.6Aa
4°C / 1 weeks	181.4 ± 1.1Babcd	187.5 ± 2.0Aa	188.6 ± 2.4Aabcd	189.2 ± 2.9Aa
4°C / 2 weeks	168.9 ± 5.4Bcd	185.9 ± 2.4Aa	182.4 ± 3.4Acd	185.6 ± 1.6Aa

[1] The symbol (⊗) referred discarded data as the pupae were emerged during cold storage treatment.

Table 8. Results of the post hoc test with Holm correction for multiple comparisons on the linear regression with parasitism rate on *Bemisia tabaci* as the response. The post hoc was performed through the R package lsmeans. Significant differences are given in bold.

Species	Contrast	Estimate	SE	df	t.ratio	Significance
Encarsia sophia	Control.10-day-old - 12C_1w.10-day-old	3.167	1.199	175	2.642	0.225
	Control.10-day-old - 12C_2w.10-day-old	19.167	1.199	175	15.992	**P < 0.001**
	Control.10-day-old - 10C_1w.10-day-old	9.667	1.199	175	8.066	**P < 0.001**
	Control.10-day-old - 10C_2w.10-day-old	19.667	1.199	175	16.409	**P < 0.001**
	Control.10-day-old - 8C_1w.10-day-old	16.917	1.199	175	14.115	**P < 0.001**
	Control.10-day-old - 8C_2w.10-day-old	20.917	1.199	175	17.452	**P < 0.001**
	Control.10-day-old - 6C_1w.10-day-old	20.417	1.199	175	17.035	**P < 0.001**
	Control.10-day-old - 6C_2w.10-day-old	22.667	1.199	175	18.912	**P < 0.001**
	Control.10-day-old - 4C_1w.10-day-old	21.417	1.199	175	17.869	**P < 0.001**
	Control.10-day-old - 4C_2w.10-day-old	25.238	1.315	175	19.199	**P < 0.001**
	Control.10-day-old - Control.12-day-old	-3.333	1.23	175	-2.711	0.192
	12C_1w.10-day-old - 12C_2w.10-day-old	16	1.167	175	13.716	**P < 0.001**
	12C_1w.10-day-old - 10C_1w.10-day-old	6.5	1.167	175	5.572	**P < 0.001**
	12C_1w.10-day-old - 10C_2w.10-day-old	16.5	1.167	175	14.144	**P < 0.001**
	12C_1w.10-day-old - 8C_1w.10-day-old	13.75	1.167	175	11.787	**P < 0.001**
	12C_1w.10-day-old - 8C_2w.10-day-old	17.75	1.167	175	15.216	**P < 0.001**
	12C_1w.10-day-old - 6C_1w.10-day-old	17.25	1.167	175	14.787	**P < 0.001**
	12C_1w.10-day-old - 6C_2w.10-day-old	19.5	1.167	175	16.716	**P < 0.001**
	12C_1w.10-day-old - 4C_1w.10-day-old	18.25	1.167	175	15.645	**P < 0.001**
	12C_1w.10-day-old - 4C_2w.10-day-old	22.071	1.285	175	17.17	**P < 0.001**
	12C_1w.10-day-old - 12C_1w.12-day-old	-5.75	1.167	175	-4.929	**P < 0.001**
	12C_2w.10-day-old - 10C_1w.10-day-old	-9.5	1.167	175	-8.144	**P < 0.001**
	12C_2w.10-day-old - 10C_2w.10-day-old	0.5	1.167	175	0.429	1
	12C_2w.10-day-old - 8C_1w.10-day-old	-2.25	1.167	175	-1.929	1
	12C_2w.10-day-old - 8C_2w.10-day-old	1.75	1.167	175	1.5	1
	12C_2w.10-day-old - 6C_1w.10-day-old	1.25	1.167	175	1.072	1
	12C_2w.10-day-old - 6C_2w.10-day-old	3.5	1.167	175	3	0.093
	12C_2w.10-day-old - 4C_1w.10-day-old	2.25	1.167	175	1.929	1
	12C_2w.10-day-old - 4C_2w.10-day-old	6.071	1.285	175	4.723	**P < 0.001**
	10C_1w.10-day-old - 10C_2w.10-day-old	10	1.167	175	8.572	**P < 0.001**
	10C_1w.10-day-old - 8C_1w.10-day-old	7.25	1.167	175	6.215	**P < 0.001**
**Species**	**Contrast**	**Estimate**	**SE**	**df**	**t.ratio**	**Significance**
	10C_1w.10-day-old - 8C_2w.10-day-old	11.25	1.167	175	9.644	**P < 0.001**

	10C_1w.10-day-old - 6C_1w.10-day-old	10.75	1.167	175	9.215	**P < 0.001**
	10C_1w.10-day-old - 6C_2w.10-day-old	13	1.167	175	11.144	**P < 0.001**
	10C_1w.10-day-old - 4C_1w.10-day-old	11.75	1.167	175	10.073	**P < 0.001**
	10C_1w.10-day-old - 4C_2w.10-day-old	15.571	1.285	175	12.113	**P < 0.001**
	10C_1w.10-day-old - 10C_1w.12-day-old	-6	1.167	175	-5.143	**P < 0.001**
	8C_1w.10-day-old - 8C_2w.10-day-old	4	1.167	175	3.429	**0.025**
	8C_1w.10-day-old - 6C_1w.10-day-old	3.5	1.167	175	3	0.093
	8C_1w.10-day-old - 6C_2w.10-day-old	5.75	1.167	175	4.929	**P < 0.001**
	8C_1w.10-day-old - 4C_1w.10-day-old	4.5	1.167	175	3.858	**0.006**
	8C_1w.10-day-old - 4C_2w.10-day-old	8.321	1.285	175	6.473	**P < 0.001**
	8C_1w.10-day-old - 8C_1w.12-day-old	-1.75	1.167	175	-1.5	1
	8C_2w.10-day-old - 6C_1w.10-day-old	-0.5	1.167	175	-0.429	1
	8C_2w.10-day-old - 6C_2w.10-day-old	1.75	1.167	175	1.5	1
	8C_2w.10-day-old - 4C_1w.10-day-old	0.5	1.167	175	0.429	1
	8C_2w.10-day-old - 4C_2w.10-day-old	4.321	1.285	175	3.362	**0.03**
	8C_2w.10-day-old - 8C_2w.12-day-old	-0.75	1.167	175	-0.643	1
	6C_1w.10-day-old - 4C_1w.10-day-old	1	1.167	175	0.857	1
	6C_1w.10-day-old - 4C_2w.10-day-old	4.821	1.285	175	3.751	**0.008**
	6C_1w.10-day-old - 6C_1w.12-day-old	-1.75	1.167	175	-1.5	1
	6C_2w.10-day-old - 4C_1w.10-day-old	-1.25	1.167	175	-1.072	1
	6C_2w.10-day-old - 4C_2w.10-day-old	2.571	1.285	175	2	1
	6C_2w.10-day-old - 6C_2w.12-day-old	-0.25	1.167	175	-0.214	1
	4C_1w.10-day-old - 4C_2w.10-day-old	3.821	1.285	175	2.973	0.093
	4C_1w.10-day-old - 4C_1w.12-day-old	-1.5	1.167	175	-1.286	1
	4C_2w.10-day-old - 4C_2w.12-day-old	-0.321	1.285	175	-0.25	1
	Control.12-day-old - 12C_1w.12-day-old	0.75	1.199	175	0.626	1
	Control.12-day-old - 10C_1w.12-day-old	7	1.199	175	5.841	P < 0.001
	Control.12-day-old - 8C_1w.12-day-old	18.5	1.199	175	15.436	P < 0.001
	Control.12-day-old - 8C_2w.12-day-old	23.5	1.199	175	19.608	P < 0.001
	Control.12-day-old - 6C_1w.12-day-old	22	1.199	175	18.356	P < 0.001
	Control.12-day-old - 6C_2w.12-day-old	25.75	1.199	175	21.485	P < 0.001
	Control.12-day-old - 4C_1w.12-day-old	23.25	1.199	175	19.399	P < 0.001
	Control.12-day-old - 4C_2w.12-day-old	28.25	1.199	175	23.571	P < 0.001
	12C_1w.12-day-old - 10C_1w.12-day-old	6.25	1.167	175	5.358	P < 0.001
**Species**	**Contrast**	**Estimate**	**SE**	**df**	**t.ratio**	**Significance**
	12C_1w.12-day-old - 8C_1w.12-day-old	17.75	1.167	175	15.216	P < 0.001
	12C_1w.12-day-old - 8C_2w.12-day-old	22.75	1.167	175	19.502	P < 0.001

	12C_1w.12-day-old - 6C_1w.12-day-old	21.25	1.167	175	18.216	P < 0.001
	12C_1w.12-day-old - 6C_2w.12-day-old	25	1.167	175	21.431	P < 0.001
	12C_1w.12-day-old - 4C_1w.12-day-old	22.5	1.167	175	19.288	P < 0.001
	12C_1w.12-day-old - 4C_2w.12-day-old	27.5	1.167	175	23.574	P < 0.001
	10C_1w.12-day-old - 8C_1w.12-day-old	11.5	1.167	175	9.858	P < 0.001
	10C_1w.12-day-old - 8C_2w.12-day-old	16.5	1.167	175	14.144	P < 0.001
	10C_1w.12-day-old - 6C_1w.12-day-old	15	1.167	175	12.859	P < 0.001
	10C_1w.12-day-old - 6C_2w.12-day-old	18.75	1.167	175	16.073	P < 0.001
	10C_1w.12-day-old - 4C_1w.12-day-old	16.25	1.167	175	13.93	P < 0.001
	10C_1w.12-day-old - 4C_2w.12-day-old	21.25	1.167	175	18.216	P < 0.001
	8C_1w.12-day-old - 8C_2w.12-day-old	5	1.167	175	4.286	0.001
	8C_1w.12-day-old - 6C_1w.12-day-old	3.5	1.167	175	3	0.093
	8C_1w.12-day-old - 6C_2w.12-day-old	7.25	1.167	175	6.215	P < 0.001
	8C_1w.12-day-old - 4C_1w.12-day-old	4.75	1.167	175	4.072	0.003
	8C_1w.12-day-old - 4C_2w.12-day-old	9.75	1.167	175	8.358	P < 0.001
	8C_2w.12-day-old - 6C_1w.12-day-old	-1.5	1.167	175	-1.286	1
	8C_2w.12-day-old - 6C_2w.12-day-old	2.25	1.167	175	1.929	1
	8C_2w.12-day-old - 4C_1w.12-day-old	-0.25	1.167	175	-0.214	1
	8C_2w.12-day-old - 4C_2w.12-day-old	4.75	1.167	175	4.072	0.003
	6C_1w.12-day-old - 6C_2w.12-day-old	3.75	1.167	175	3.215	0.048
	6C_1w.12-day-old - 4C_1w.12-day-old	1.25	1.167	175	1.072	1
	6C_1w.12-day-old - 4C_2w.12-day-old	6.25	1.167	175	5.358	P < 0.001
	6C_2w.12-day-old - 4C_1w.12-day-old	-2.5	1.167	175	-2.143	0.804
	6C_2w.12-day-old - 4C_2w.12-day-old	2.5	1.167	175	2.143	0.804
	4C_1w.12-day-old - 4C_2w.12-day-old	5	1.167	175	4.286	0.001
Eretmocerus hayati	Control - 12C_1w	6	1.131	179	5.305	P < 0.001
	Control - 10C_1w	6.5	1.131	179	5.747	P < 0.001
	Control - 10C_2w	17.667	1.411	179	12.523	P < 0.001
	Control - 8C_1w	13.25	1.131	179	11.715	P < 0.001
	Control - 8C_2w	20.25	1.131	179	17.903	P < 0.001
	Control - 6C_1w	19.625	1.131	179	17.351	P < 0.001
	Control - 6C_2w	27.5	1.131	179	24.313	P < 0.001
	Control - 4C_1w	26.25	1.131	179	23.208	P < 0.001
**Species**	**Contrast**	**Estimate**	**SE**	**df**	**t.ratio**	**Significance**
	Control - 4C_2w	30.375	1.131	179	26.855	P < 0.001
	12C_1w - 10C_1w	0.5	1.131	179	0.442	1
	12C_1w - 10C_2w	11.667	1.411	179	8.27	P < 0.001

	12C_1w - 8C_1w	7.25	1.131	179	6.41	P < 0.001
	12C_1w - 8C_2w	14.25	1.131	179	12.599	P < 0.001
	12C_1w - 6C_1w	13.625	1.131	179	12.046	P < 0.001
	12C_1w - 6C_2w	21.5	1.131	179	19.008	P < 0.001
	12C_1w - 4C_1w	20.25	1.131	179	17.903	P < 0.001
	12C_1w - 4C_2w	24.375	1.131	179	21.55	P < 0.001
	10C_1w - 10C_2w	11.167	1.411	179	7.916	P < 0.001
	10C_1w - 8C_1w	6.75	1.131	179	5.968	P < 0.001
	10C_1w - 8C_2w	13.75	1.131	179	12.157	P < 0.001
	10C_1w - 6C_1w	13.125	1.131	179	11.604	P < 0.001
	10C_1w - 6C_2w	21	1.131	179	18.566	P < 0.001
	10C_1w - 4C_1w	19.75	1.131	179	17.461	P < 0.001
	10C_1w - 4C_2w	23.875	1.131	179	21.108	P < 0.001
	10C_2w - 8C_1w	-4.417	1.411	179	-3.131	0.014
	10C_2w - 8C_2w	2.583	1.411	179	1.831	0.344
	10C_2w - 6C_1w	1.958	1.411	179	1.388	0.667
	10C_2w - 6C_2w	9.833	1.411	179	6.971	P < 0.001
	10C_2w - 4C_1w	8.583	1.411	179	6.084	P < 0.001
	10C_2w - 4C_2w	12.708	1.411	179	9.009	P < 0.001
	8C_1w - 8C_2w	7	1.131	179	6.189	P < 0.001
	8C_1w - 6C_1w	6.375	1.131	179	5.636	P < 0.001
	8C_1w - 6C_2w	14.25	1.131	179	12.599	P < 0.001
	8C_1w - 4C_1w	13	1.131	179	11.493	P < 0.001
	8C_1w - 4C_2w	17.125	1.131	179	15.14	P < 0.001
	8C_2w - 6C_1w	-0.625	1.131	179	-0.553	1
	8C_2w - 6C_2w	7.25	1.131	179	6.41	P < 0.001
	8C_2w - 4C_1w	6	1.131	179	5.305	P < 0.001
	8C_2w - 4C_2w	10.125	1.131	179	8.952	P < 0.001
	6C_1w - 6C_2w	7.875	1.131	179	6.962	P < 0.001
	6C_1w - 4C_1w	6.625	1.131	179	5.857	P < 0.001
	6C_1w - 4C_2w	10.75	1.131	179	9.504	P < 0.001
	6C_2w - 4C_1w	-1.25	1.131	179	-1.105	0.812
**Species**	**Contrast**	**Estimate**	**SE**	**df**	**t.ratio**	**Significance**
	6C_2w - 4C_2w	2.875	1.131	179	2.542	0.071
	4C_1w - 4C_2w	4.125	1.131	179	3.647	0.003

Table S9. Effect of cold storage on sex ratio of emerged *Eretmocerus hayati* after the pupae exposed to different temperatures and duration. Data are means ± SE.

Storage treatment	Sex ratio (proportion of males)[a]	
	12-day-old pupae	15-day-old pupae
Control	0.47 ± 0.03a	0.47 ± 0.02a
12°C / 1 week	0.47 ± 0.05a	0.47 ± 0.04a
10°C / 1 week	0.45 ± 0.02a	0.46 ± 0.03a
10°C / 2 weeks	0.49 ± 0.06a	⊗ [1]
8°C / 1 week	0.43 ± 0.05a	0.48 ± 0.03a
8°C / 2 weeks	0.45 ± 0.05a	0.47 ± 0.05a
6°C / 1 week	0.46 ± 0.08a	0.47 ± 0.03a
6°C / 2 weeks	0.47 ± 0.09a	0.48 ± 0.05a
4°C / 1 week	0.45 ± 0.08a	0.39 ± 0.08a
4°C / 2 weeks	0.55 ± 0.06a	0.49 ± 0.07a

[1] The symbol (⊗) referred discarded data as the pupae were emerged during cold storage treatment.
